# Effects of exercise interventions on cognitive function in sedentary adults: a systematic review and network meta-analysis

**DOI:** 10.3389/fpubh.2026.1794345

**Published:** 2026-05-14

**Authors:** Yanyan Shao, Xingnian Long

**Affiliations:** 1 School of Wushu, Wuhan Sports University, Wuhan, China; 2 The College of Sports Science and Technology of Wuhan Sports University, Wuhan, China

**Keywords:** cognitive function, exercise, network meta-analysis, sedentary behavior, systematic review

## Abstract

**Objective:**

This study aims to systematically compare and evaluate the effects of different types of exercise interventions on cognitive function in sedentary adults through a systematic review and network meta-analysis, quantifying their relative effectiveness to provide evidence for formulating precise exercise prescriptions.

**Methods:**

Electronic databases including PubMed, Embase, Cochrane Library, Scopus, Web of Science, and EBSCO were systematically searched from their inception until December 30, 2025, for RCTs comparing exercise interventions with a sedentary control on cognitive outcomes in sedentary adults. Literature screening, data extraction, and risk-of-bias assessment were conducted based on predefined PICOS criteria. For global cognition and executive function, which involved multiple exercise types forming connected networks, a frequentist network meta-analysis was performed to integrate direct and indirect evidence. The surface under the cumulative ranking curve was used to rank interventions. For memory function, due to the absence of a connected network, a standard pairwise meta-analysis was conducted using a random-effects model. All effect sizes were calculated as standardized mean differences with 95% confidence intervals.

**Results:**

Seventeen RCTs involving 2,187 sedentary adults were included. Interventions were categorized as aerobic exercise, resistance exercise, and multicomponent exercise. The network meta-analysis results indicated that for improving global cognitive function, multicomponent exercise was most effective (SUCRA = 88.5%), followed by aerobic exercise (SUCRA = 58.3%) and resistance exercise (SUCRA = 48.5%). For executive function, aerobic exercise ranked highest (SUCRA = 90.8%), followed by multicomponent exercise (SUCRA = 50.7%) and resistance exercise (SUCRA = 38.1%). For memory function, exercise demonstrated a small-to-moderate improvement compared to control that approached but did not reach statistical significance (SMD = 0.33, 95% CI: −0.02 to 0.68). According to the GRADE framework, direct comparisons were rated as moderate quality, while most indirect and mixed comparisons showed very low certainty, indicating the need for cautious interpretation.

**Conclusion:**

Exercise interventions effectively improve cognitive function in sedentary adults, with effects demonstrating domain specificity. Multicomponent exercise appears optimal for enhancing global cognition, while aerobic exercise shows prominent benefits for improving executive function. These findings support targeting exercise prescriptions to specific cognitive goals, although further high-quality research is warranted for confirmation.

**Systematic review registration:**

https://www.crd.york.ac.uk/PROSPERO/view/CRD420251253097, identifier PROSPERO (CRD420251253097).

## 1 Introduction

With the profound transformation of work and lifestyle in modern society, sedentary behavior has become an increasingly prominent global public health issue (1). Prolonged sitting is significantly associated with the risk of various chronic diseases. Beyond its negative impacts on cardiovascular and metabolic health (2, 3), mounting evidence suggests that a sedentary lifestyle may pose a threat to brain health, correlating with cognitive decline, diminished executive function, and even an increased risk of neurodegenerative diseases (4–9). In this context, determining how to effectively improve the cognitive health of sedentary populations through interventions has become a critical issue urgently needing to be addressed in the fields of exercise science and public health.

Exercise intervention, as a core strategy for improving both physical and mental well-being, has garnered widespread research support for its cognitive benefits (10, 11). Existing evidence indicates that regular physical activity can promote brain structural plasticity, optimize neurometabolism, and enhance the efficiency of neural network connectivity, thereby improving various cognitive abilities, including memory, attention, and executive function (10, 12–15). However, exercise is not a homogeneous intervention; variations in its type, intensity, frequency, and duration can lead to markedly different neurocognitive effects (16, 17). For instance, aerobic exercise may primarily function by enhancing cardiovascular function and cerebral blood flow (18, 19), whereas resistance training might be more closely related to the regulation of neurotrophic factors and neuromuscular adaptation (20, 21). Furthermore, mind–body exercises (e.g., yoga, Tai Chi) are also believed to potentially influence cognition by modulating stress responses and attentional control mechanisms (22, 23). Nevertheless, there remains a lack of systematic, quantitative comparison and conclusive evidence regarding which specific type of exercise is most effective for improving cognitive function in sedentary adults.

Current research in this field suffers from several key limitations. First, most randomized controlled trials focus on comparing a single exercise mode against a sedentary control, lacking direct comparisons between different exercise types. Second, while traditional meta-analyses can synthesize the overall effect of exercise versus no exercise, they are limited in their ability to parse the relative merits of different exercise subtypes. Finally, the sedentary population is heterogeneous, with potential differences in cognitive baseline, physiological characteristics, and exercise adaptability, necessitating more refined effect evaluations. These knowledge gaps constrain the development of personalized and precise exercise prescriptions in clinical and public health practice.

Network meta-analysis, an advanced evidence synthesis method, can simultaneously integrate direct and indirect comparative evidence to rank and quantify the relative effects of multiple interventions. Applying this method to this field holds the promise of overcoming the fragmented nature of existing research, systematically evaluating and comparing the differential effects of various exercise modalities on different dimensions of cognitive function in sedentary adults, thereby providing high-level evidence to support the optimization of exercise prescriptions.

Therefore, this study aims to conduct a systematic review and network meta-analysis to comprehensively assess the improving effects of different types of exercise interventions [including aerobic exercise (AE), resistance exercise (RE), and multicomponent exercise (ME)] on the cognitive function of sedentary adults and to quantify their relative effectiveness. The findings are expected to provide key scientific evidence for formulating public health guidelines, implementing clinical exercise prescriptions, and developing individualized health promotion strategies.

## 2 Methods

### 2.1 Study protocol and registration

This meta-analysis was conducted following the Preferred Reporting Items for Systematic Reviews and Meta-Analyses (PRISMA) guidelines, its extension for network meta-analyses (PRISMA-NMA), and the Cochrane Handbook for Systematic Reviews of Interventions. The study protocol was pre-registered on PROSPERO (Registration number: CRD420251253097). As this study involved secondary data analysis without direct human subject contact, institutional review board approval was not required.

### 2.2 Literature search strategy

To comprehensively identify relevant studies, we systematically searched the following electronic databases: PubMed, Embase, Cochrane Central Register of Controlled Trials, Scopus, Web of Science, and EBSCO. The search timeframe was from database inception to December 30, 2025, with no language restrictions. The search strategy combined subject headings and free-text terms around three core concepts: Population (e.g., “sedentary behavior,” “sedentary lifestyle”), Intervention (e.g., “Exercise,” “Physical Activity,” “Aerobic Exercises,” “Resistance Training,” “Yoga”), and Outcome (e.g., “cognition,” “cognitive function”). Detailed search strategies for each database are provided in the Supplementary file S1.

Two independent reviewers (Yy-S and Xn-L) screened records based on title and abstract, followed by full-text assessment against the inclusion criteria. Disagreements were resolved through consensus. EndNote X9 software was used for screening and management of records. Additionally, reference lists of included studies and relevant reviews were manually searched to identify any missed publications.

### 2.3 Inclusion and exclusion criteria

The PICOS framework was used to define the inclusion criteria:

Population (P): This review will include adults aged 18 years or older who exhibit sedentary behavior. This review defines “sedentary adults” as individuals who meet any of the following criteria at baseline: engaging in less than 150 min of moderate-intensity physical activity per week, accumulating less than 60 min of moderate-to-vigorous physical activity per week, failing to meet established physical activity guidelines for a minimum of 3–6 months prior to study enrollment, exercising less than twice per week on average over the previous 5 years, or scoring low on validated physical activity questionnaires. Eligible participants encompass both generally healthy individuals and those with cardiometabolic risk factors (such as overweight or prediabetes), provided they have no major cognitive impairment. Individuals will be excluded if they have a diagnosed neurodegenerative disease (e.g., dementia, Parkinson’s disease), a severe psychiatric disorder, or any other condition that would preclude safe participation in an exercise program.

Intervention (I): To facilitate analysis, all exercise types were categorized into three categories based on a previous classification (24): AE (e.g., walking, jogging, cycling, high-intensity interval training, moderate-intensity continuous training), RE, and ME.

Comparison (C): The control group was not allowed to receive any other interventions that could affect cognitive function.

Outcome (O): The main outcome measures were scores from cognitive function assessment scales.

Study Design (S): Only randomized controlled trials (RCTs) were included. Conference abstracts, reviews, letters, case reports, studies assessing only acute effects of single exercise sessions, and animal studies were excluded.

Studies were excluded if they: (1) lacked an appropriate control group, (2) were conference proceedings, protocols, or systematic reviews, (3) had inappropriate interventions, or (4) had unavailable data.

### 2.4 Data extraction and synthesis

Data were extracted independently by two reviewers and cross-checked. Discrepancies were resolved through discussion. Extracted information included: (1) Study characteristics (author, publication year, country); (2) Participant characteristics (sample size, age); (3) Intervention and control details (exercise type, frequency, MET value, duration per session, total intervention period); (4) Outcome data (mean scores, standard deviations, and sample sizes at various time points). If data were missing or unclear, corresponding authors were contacted via email.

When a study reported multiple measures for the same cognitive domain, data extraction followed a hierarchical priority logic: we prioritized the measure explicitly designated by the authors as the primary cognitive outcome; next, standardized computerized batteries (e.g., NIH Toolbox, Cogstate) were given preference due to their validated composite scores and minimized practice effects; domain-specific tests were prioritized based on established sensitivity to exercise interventions (e.g., Stroop interference, Flanker, or WCST for executive function; CVLT or RAVLT for memory; MoCA or 3MSE for global cognition); if multiple tests were reported without a primary outcome, the most frequently used test across the literature or the one with the largest effect size was extracted; and when both individual tests and a domain composite score were reported, the composite score was prioritized.

### 2.5 Intervention classification

To systematically compare the effects of different exercise modalities on cognitive function, all exercise interventions from the included studies were categorized into three distinct types based on previously established classification frameworks (24) and their physiological characteristics: AE, RE, and ME. AE was defined as rhythmic, continuous activities engaging large muscle groups aimed at improving cardiorespiratory fitness, including walking, jogging, swimming, and cycling. RE involved exercises against external resistance to enhance muscular strength and endurance. ME comprised combined training programs integrating two or more exercise modalities, such as aerobic plus resistance training, or incorporating mind–body elements.

### 2.6 Risk of bias and evidence quality assessment

Two independent reviewers (Yy-S and Xn-L) assessed the risk of bias of included studies using the Cochrane Risk of Bias 2.0 (RoB 2.0) tool. Any disagreements were resolved through consensus. The Grading of Recommendations Assessment, Development and Evaluation (GRADE) system was used to assess certainty separately for direct, indirect, and mixed comparisons.

### 2.7 Pairwise meta-analysis

A paired meta-analysis was first conducted to examine the effect differences between various exercise interventions and control groups, respectively. Effect sizes were estimated with the standardized mean differences (SMDs) and 95% confidence intervals (95% CIs) using change-from-baseline values by the random effects model.

To explore potential sources of clinical heterogeneity, subgroup analyses were conducted for the pairwise meta-analysis, stratified by exercise type (AE, RE and ME). For comparisons with sufficient studies, additional subgroup analyses were performed based on intervention duration (<24 weeks vs. ≥24 weeks) and participant age. For participant age, the median age of all included studies was 68 years, and this value was therefore used as the cut-off to divide studies into younger (<68 years) and older (≥68 years) subgroups. This cut-off balanced the number of studies across subgroups and avoided arbitrary threshold selection. Due to the limited number of studies for formal meta-regression, exploratory analyses were conducted using bubble plots to visually assess the relationship between effect size and two continuous variables: intervention duration and mean participant age. Bubble plots were generated separately for each cognitive outcome, where the size of each bubble represented the study weight. Subgroup analyses with small study numbers and all bubble plots should be interpreted as exploratory. All analyses were conducted using Stata 17.0.

### 2.8 Network meta-analysis

To minimize the impact of baseline differences, change-from-baseline values (mean change and standard deviation) were used to synthesize effect sizes. The formula provided in the Cochrane Handbook was used to calculate the standard deviation of change. Given the variety of scales used to assess global cognition function and executive function, SMD was used as the effect measure. Network plots were generated using Stata 17.0 to illustrate the relationships between interventions, where lines connecting nodes represent direct comparisons, line thickness is proportional to the number of studies, and node size is proportional to the sample size. Loop inconsistency factors and their 95% confidence intervals were calculated to evaluate inconsistency within each closed loop. Inconsistency models were used to detect any inconsistency; when the *p*-value was >0.05, a consistency model was applied for analysis. The surface under the cumulative ranking curve (SUCRA) was used to rank and compare the effectiveness of different exercise interventions, with SUCRA values ranging from 0 to 100% (higher values indicating better effectiveness). Funnel plots were used to examine potential publication bias or small-study effects.

## 3 Results

### 3.1 Literature screening process

**Figure 1:**
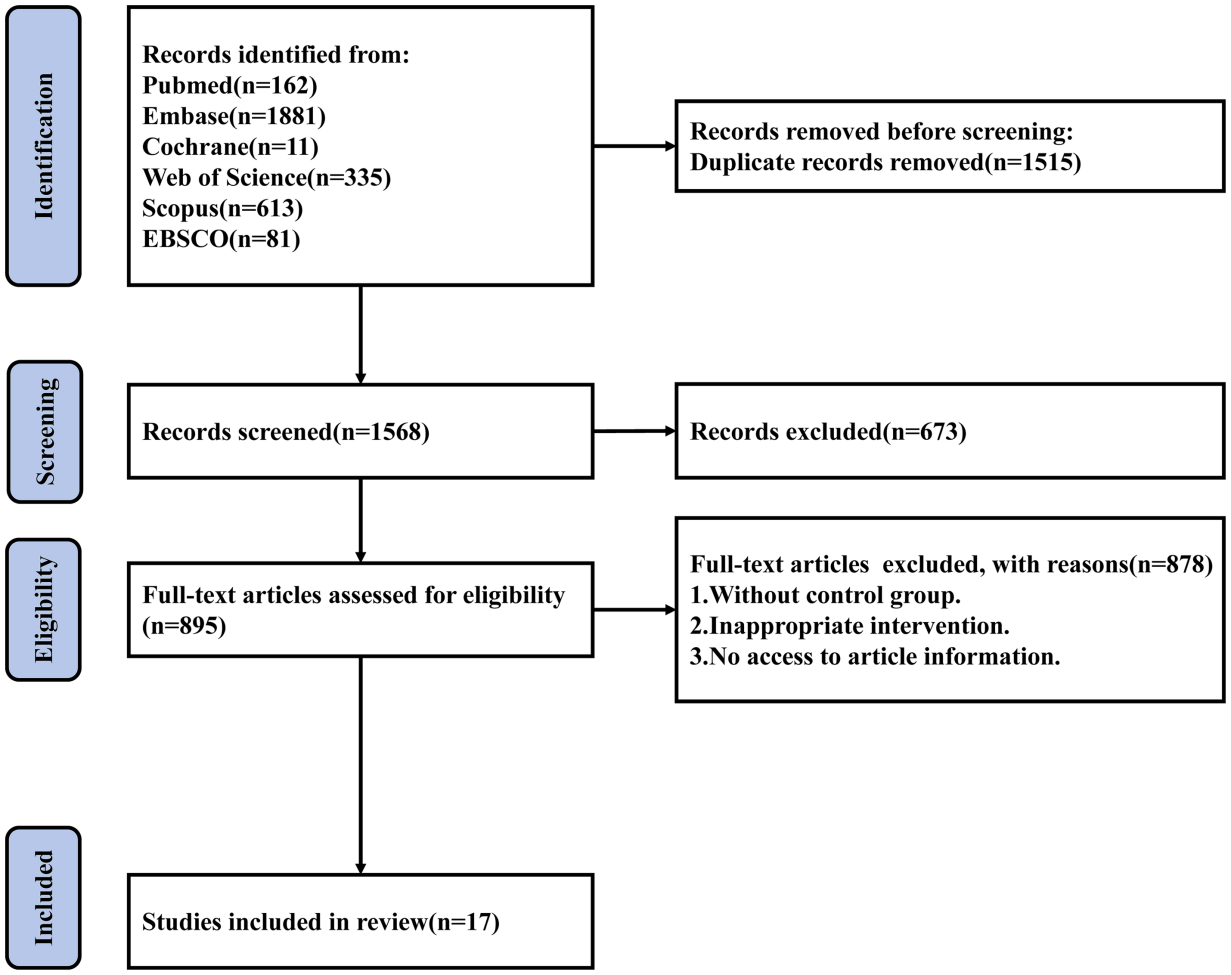
Flowchart of literature screening.

The systematic database search initially yielded 3,083 records. After removing duplicates, 1,568 unique publications remained. Screening of titles and abstracts excluded 673 clearly irrelevant studies. Full texts of the remaining 895 articles were retrieved and reviewed in detail. Ultimately, 17 RCTs met the pre-defined inclusion criteria and were included in this systematic review and meta-analysis (25–41). The detailed screening process and reasons for exclusion are shown in Figure 1.

### 3.2 Characteristics of included studies

The 17 studies involved a total of 2,187 sedentary adults. Sample size of included studies ranged from 17 to 1,476 participants per study. The mean age of participants ranged from 21.62 to 82.4 years. Exercise types included AE, RE, and ME. Specifically, 13 studies involved AE, 2 involved RE, and 4 involved ME. Exercise frequency ranged from 2 to 7 sessions per week. Session duration ranged from 20 to 65 min. All studies reported outcome measures for global cognition function and/or executive function and/or memory function. Details are provided in the Supplementary file S3.

### 3.3 Risk of bias assessment results

**Figure 2:**
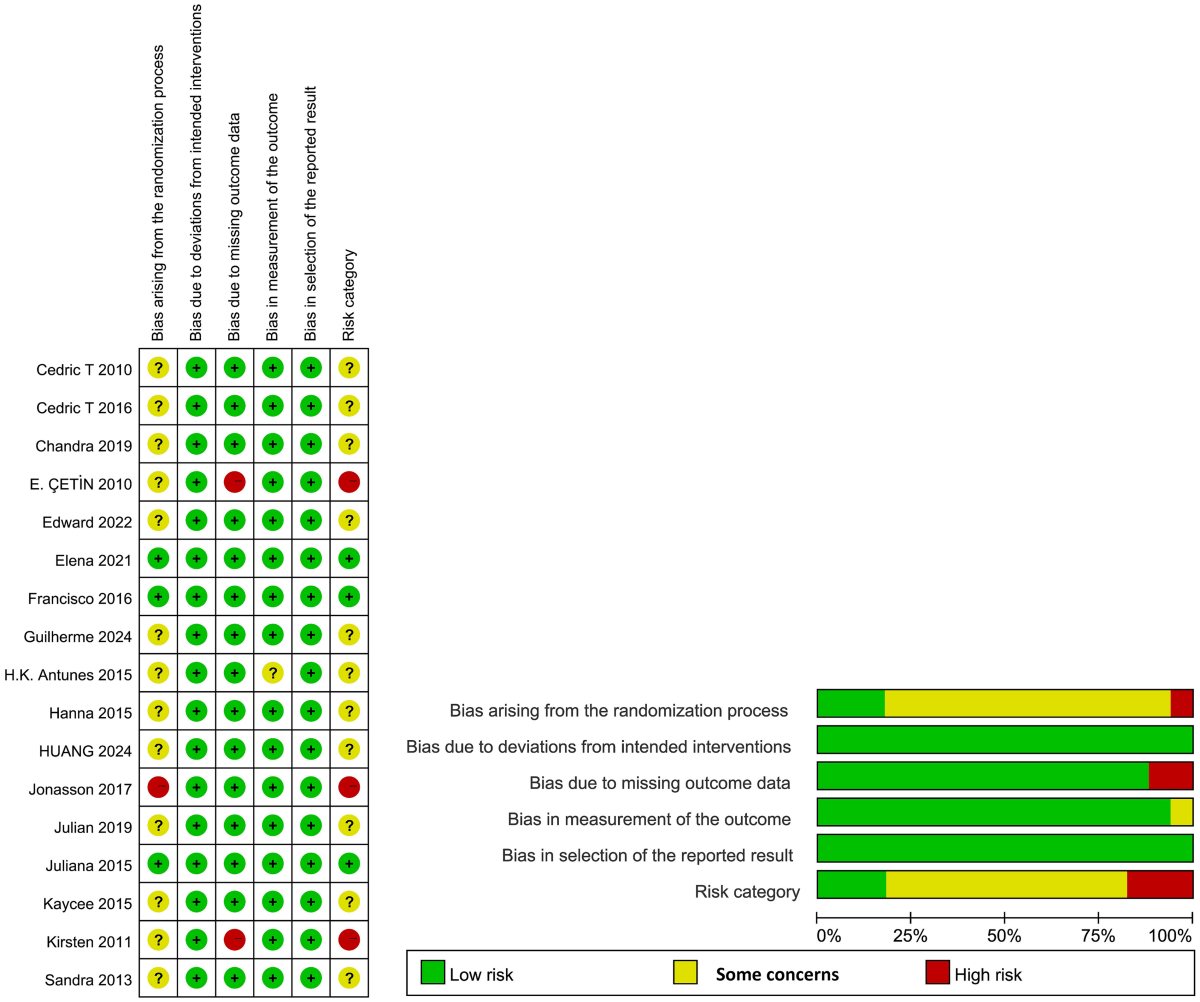
Risk of bias assessment results.

Assessment using the RoB 2.0 tool showed: regarding the randomization process, 13 studies (76.5%) raised “some concerns” and 1 study (5.9%) raised “High risk”; regarding deviations from intended interventions, all 17 studies (100%) were judged “low risk”; regarding missing outcome data, 2 study (11.8%) was “high risk”; regarding measurement of the outcome, 1 study (5.9%) was “some concerns”; regarding selection of the reported result, all 17 studies (100%) were “low risk.” Overall, 3 studies (17.65%) were judged “low risk,” 11 (64.7%) as “some concerns,” and 3 (17.65%) as “high risk.” Details are shown in Figure 2.

### 3.4 Pairwise meta-analysis

A pairwise meta-analysis was first conducted to provide an overview of the direct evidence and to examine the overall effect of exercise interventions compared to control for each cognitive outcome. Due to the different evidence structures, the results for memory function are presented as the primary pairwise analysis, while the results for global cognition and executive function serve as descriptive context for the subsequent network meta-analysis.

#### 3.4.1 Memory function

For memory function, although studies involving AE versus control and ME versus control were identified, the evidence network remained disconnected due to the absence of direct comparisons between AE and ME. Consequently, a network meta-analysis was not feasible. A pairwise meta-analysis was therefore conducted to pool all available studies comparing exercise interventions (AE and ME) with control, which included 8 studies with a total of 1797 sedentary adults.

**Figure 3:**
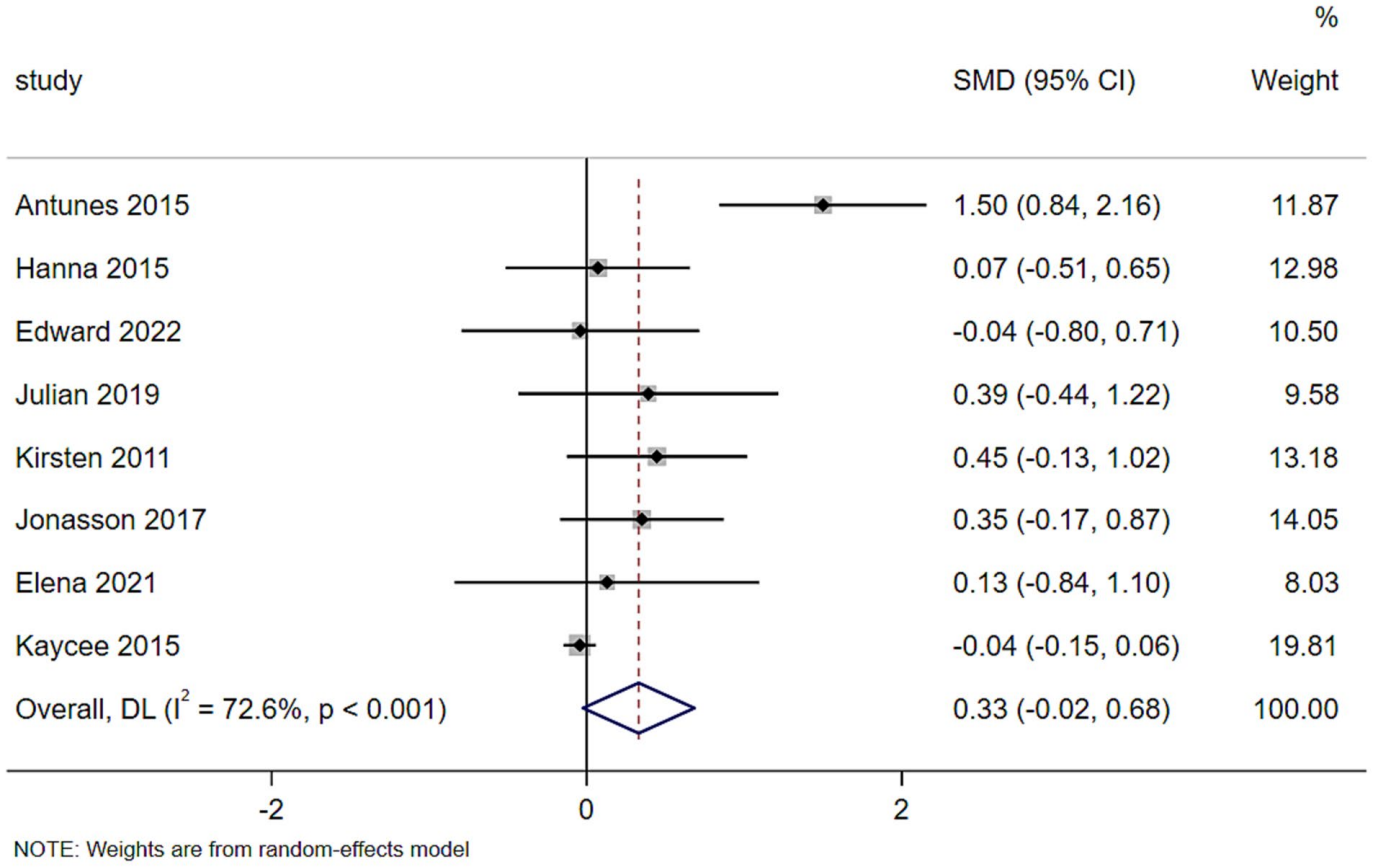
Forest plot of the effect of exercise on memory function in sedentary adults.

The pooled results showed that exercise improved memory function compared to control, with a SMD of 0.33 (95% CI: −0.02 to 0.68), indicating a small-to-moderate effect that approached but did not reach statistical significance. However, substantial heterogeneity was observed (*I*^2^ = 72.6%, *τ*^2^ = 0.1451, *p* < 0.001), suggesting considerable variability among the included studies (Figure 3).

To explore the sources of heterogeneity, a sensitivity analysis was performed by omitting one study at a time. Exclusion of the LIFE trial (40)—the largest study in this analysis (weight = 19.81%)—resulted in a substantial reduction in heterogeneity (*I*^2^ = 55.9%) and a slightly larger effect size that reached statistical significance (SMD = 0.423, 95% CI: 0.04 to 0.806). This suggests that the LIFE trial contributed substantially to the observed heterogeneity. Exclusion of any other single study did not materially alter the pooled effect size or heterogeneity. Given that the LIFE trial met all prespecified inclusion criteria, it was retained in the primary analysis, and the results excluding it are presented as a sensitivity analysis in the Supplementary material (Supplementary file S5).

Subgroup analyses were performed to explore the influence of intervention duration and participant age on memory function. Due to the limited number of studies for ME (*n* = 1), subgroup analysis by exercise type was not conducted. Detailed results, including heterogeneity statistics, are presented in Supplementary file S5.

Using the median age of 68 years as the cut-off, the four studies with mean age < 68 years showed a small non-significant effect (SMD = 0.22, 95% CI: −0.11 to 0.55, *I*^2^ = 0.0%), whereas the four studies with mean age ≥ 68 years showed a larger but still non-significant effect (SMD = 0.46, 95% CI: −0.21 to 1.13, *I*^2^ = 86.7%). The difference between subgroups was not statistically significant (*p* for interaction = 0.534).

With respect to intervention duration, the difference between subgroups was not statistically significant (*p* = 0.305). Studies with ≥ 24 weeks showed high heterogeneity (*I*^2^ = 80.3%, *n* = 6) and a non-significant effect, while studies with < 24 weeks (*n* = 2) showed low heterogeneity (*I*^2^ = 0.0%) and a non-significant effect.

#### 3.4.2 Global cognitive function

For global cognitive function, pairwise meta-analysis including 9 studies demonstrated that exercise interventions significantly improved global cognition compared to control, with a moderate effect size (SMD = 0.57, 95% CI: 0.11–1.03). Low heterogeneity was observed (*I*^2^ = 34.7%, *τ*^2^ = 0.0565, *p* = 0.130).

Subgroup analyses revealed that intervention duration partially accounted for the observed heterogeneity, with a near-significant between-group difference (*p* = 0.063) and low heterogeneity within the shorter-duration subgroup (*I*^2^ = 0.0%). In contrast, exercise type and participant age did not significantly explain the heterogeneity, with non-significant interaction *p*-values (*p* = 0.335 and *p* = 0.964, respectively) and persistent moderate-to-high heterogeneity within certain subgroups. Detailed results are presented in Supplementary file S5.

#### 3.4.3 Executive function

For executive function, pairwise meta-analysis including 15 studies showed a significant benefit of exercise interventions over control (SMD = 0.27, 95% CI: 0.08 to 0.47), with moderate heterogeneity (*I*^2^ = 48.4%, *τ*^2^ = 0.0636, *p* = 0.014). The forest plot is presented in Supplementary file S5.

Subgroup analyses suggested that age partially accounted for the observed heterogeneity, with a significant between-group difference (*p* for interaction = 0.014). The eight studies with mean age < 68 years showed a moderate and statistically significant improvement (SMD = 0.50, 95% CI: 0.19 to 0.80, *I*^2^ = 42.1%), whereas the seven studies with mean age ≥ 68 years showed a negligible non-significant effect (SMD = 0.07, 95% CI: −0.08 to 0.22, *I*^2^ = 10.1%). In contrast, exercise type and intervention duration did not significantly explain the heterogeneity, with non-significant interaction *p*-values (*p* = 0.165 and *p* = 0.071, respectively) and persistent moderate heterogeneity within certain subgroups. Detailed results are presented in Supplementary file S5.

#### 3.4.4 Exploration of continuous moderators

**Figure 4:**
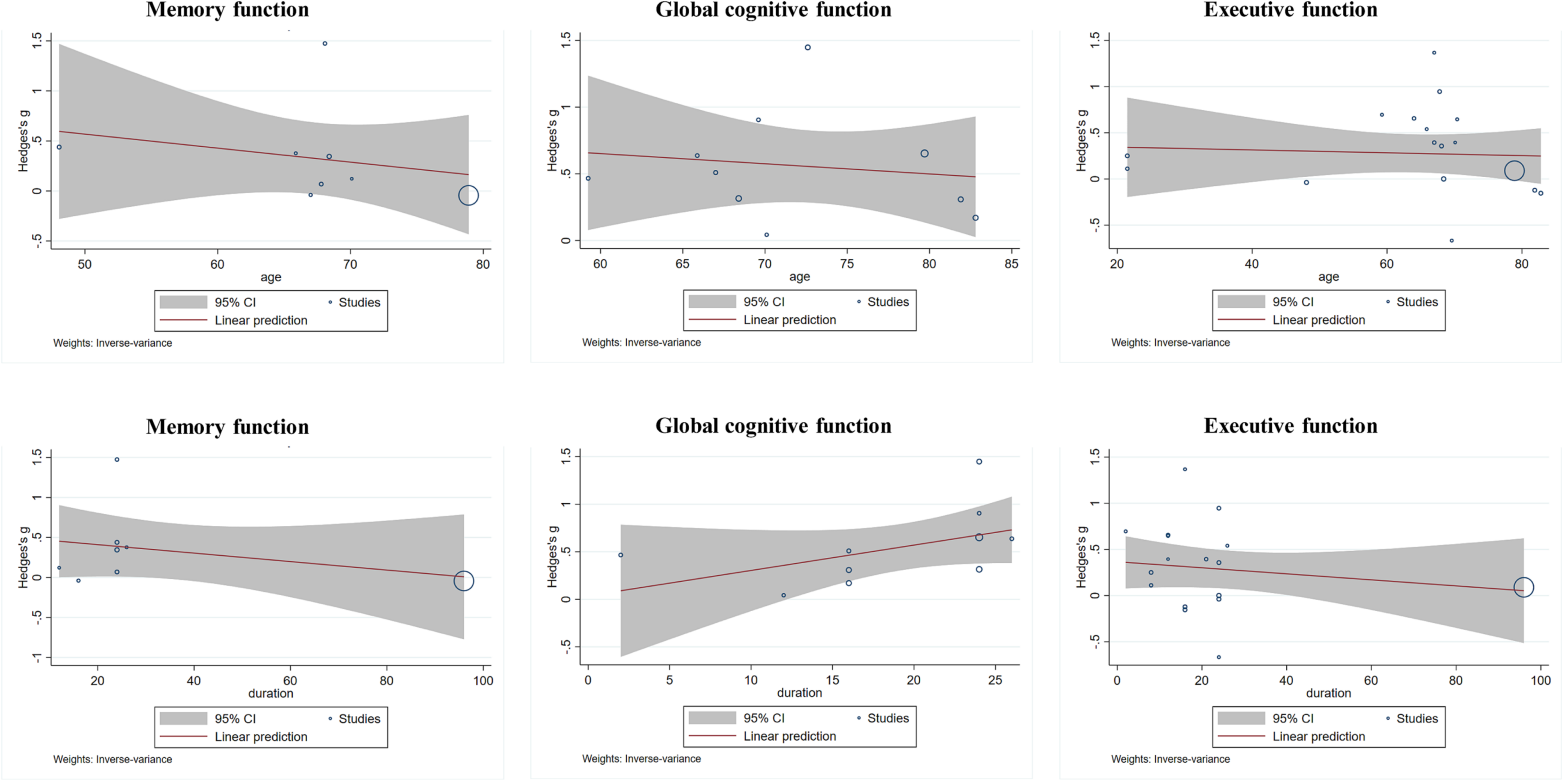
Meta-regression (bubble plots) of global cognition, memory, and executive function with age and exercise duration.

Bubble plots were generated to visually examine the relationship between effect size (Hedges’ g) and two continuous moderators (intervention duration and mean participant age) for each cognitive outcome. Across all three cognitive domains, neither intervention duration nor mean participant age showed a clear linear association with effect size. The fitted regression lines remained relatively flat across the range of included studies, suggesting no consistent dose–response relationship for either moderator. Complete bubble plots are presented in Figure 4. Due to the limited number of studies, these exploratory visual assessments should be interpreted with caution.

### 3.5 Network meta-analysis results

#### 3.5.1 Network evidence graphs

**Figure 5:**
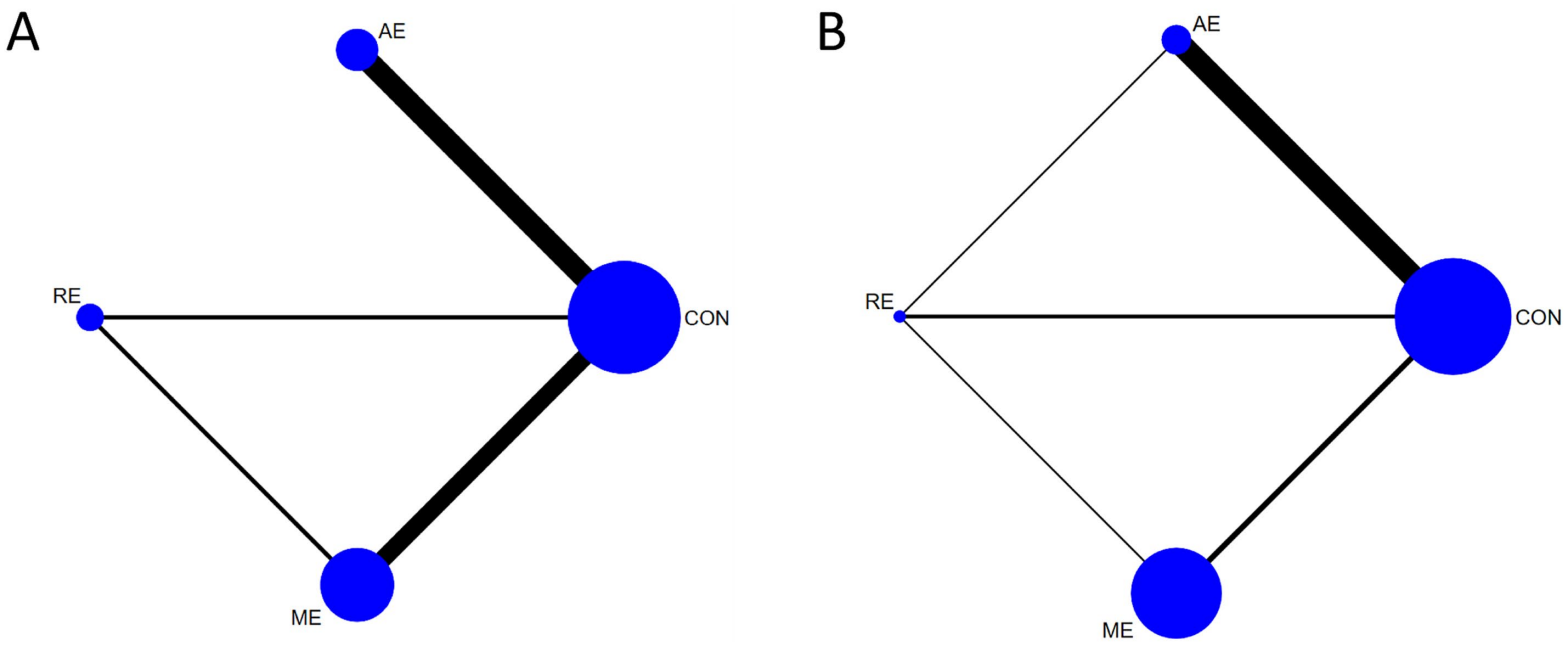
Network evidence diagram. **(A)** Network evidence diagram of the effects of different exercise intervention methods on improving global cognitive function. **(B)** Network evidence diagram of the effects of different exercise intervention methods on improving executive function. CON, control group; AE, aerobic exercise; RE, resistance exercise; ME, multicomponent exercise.

In the network graphs (Figure 5), node size is proportional to the sample size for each intervention, and the thickness of connecting lines represents the number of studies comparing the linked exercise modalities.

#### 3.5.2 Heterogeneity assessment

To evaluate between-study heterogeneity within the network, a random-effects consistency model was employed to estimate the overall heterogeneity parameters. For global cognitive function, the estimated network heterogeneity variance was *τ*^2^ = 0.0567, indicating low to moderate heterogeneity across the included studies. For executive function, the network *τ*^2^ was 0.0699, also suggesting a low to moderate degree of heterogeneity. These levels of heterogeneity are within the acceptable range for network meta-analyses and likely reflect clinical and methodological diversity among the included studies, such as variations in participant characteristics, intervention protocols, and outcome measurement tools.

#### 3.5.3 Inconsistency assessment

Inconsistency was evaluated using loop inconsistency tests, model fit comparisons, and node-splitting methods. For global cognitive function improvement, loop inconsistency tests showed good consistency for “CON-RE-ME” loop. Inconsistency model tests indicated *p*-values > 0.05 for all outcomes, suggesting no significant inconsistency; therefore, consistency models were applied. Node-splitting showed good agreement between direct and indirect evidence for all outcomes (*p* > 0.05), indicating high reliability (Supplementary file S6). For executive function improvement, loop inconsistency tests showed good consistency for “CON-AE-RE” and “CON-RE-ME” loops. Inconsistency model tests indicated *p*-values > 0.05 for all outcomes, suggesting no significant inconsistency; therefore, consistency models were applied. Node-splitting showed good agreement between direct and indirect evidence for all outcomes (*p* > 0.05), indicating high reliability (Supplementary file S6).

#### 3.5.4 SUCRA probability ranking

**Figure 6:**
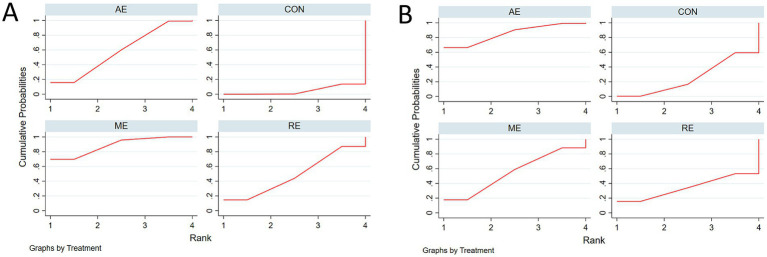
Ranking Probabilities (SUCRA Values). **(A)** SUCRA values for global cognitive function; **(B)** SUCRA values for executive function. CON, control group; AE, aerobic exercise; RE, resistance exercise; ME, multicomponent exercise.

As shown in Figure 6, SUCRA ranking results indicated that for global cognitive function, ME had the highest probability of being the most effective intervention (SUCRA = 88.5%), followed by AE (SUCRA = 58.3%), and then RE (SUCRA = 48.5%). For executive function, AE had the highest probability of being the most effective intervention (SUCRA = 90.8%), followed by multicomponent exercise (SUCRA = 50.7%) and resistance exercise (SUCRA = 38.1%).

### 3.6 Publication bias test

**Figure 7:**
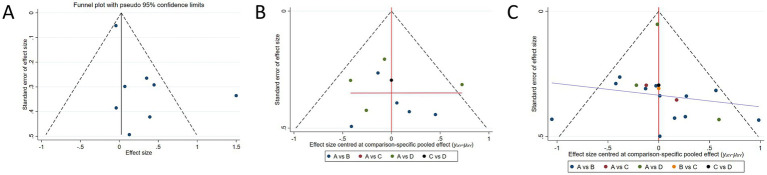
Publication bias test. **(A)** Standard funnel plot for memory function. **(B)** Comparison-adjusted funnel plot for global cognitive function: A: control group; B: aerobic exercise; C: resistance exercise; D: multicomponent exercise. **(C)** Comparison-adjusted funnel plot for executive function: A: control group; B: aerobic exercise; C: resistance exercise; D: multicomponent exercise.

Publication bias was assessed using funnel plots, Begg’s test, and Egger’s regression test. For global cognitive function, neither Begg’s test (*p* = 0.655) nor Egger’s test (*p* = 0.944) indicated significant publication bias. Similarly, no statistically significant publication bias was detected for memory function (Begg’s test: *p* = 0.881; Egger’s test: *p* = 0.866). Begg’s test suggested potential publication bias (*p* = 0.026), although Egger’s test was not significant (*p* = 0.087). Visual inspection of funnel plots revealed slight asymmetry for executive function and memory function, suggesting potential small-study effects. Therefore, the possibility of publication bias cannot be completely ruled out for these outcomes (Figure 7).

### 3.7 GRADE evidence quality assessment

The GRADE assessment (Supplementary file S7) revealed that for global cognitive function and executive function, direct comparisons were rated as moderate quality, while most indirect and mixed comparisons were rated as very low certainty, primarily due to study limitations, imprecision, and publication bias concerns. For memory function, the certainty of evidence for AE versus control was rated as moderate, and for ME versus control as low.

## 4 Discussion

This study employed a network meta-analysis to synthesize the best available evidence, providing the systematic quantification and ranking of the relative effectiveness of three common exercise intervention modalities in improving different cognitive domains among sedentary adults. The main findings reveal a distinct domain-specific effect pattern: ME had the highest probability of being the optimal intervention for enhancing global cognitive function (SUCRA = 88.5%), while AE ranked first in improving executive function (SUCRA = 90.8%). All structured exercise interventions were significantly superior to the sedentary control condition. These results provide key evidence for formulating precise exercise prescriptions targeting specific cognitive goals.

### 4.1 Interpretation of key findings and mechanistic insights

ME demonstrated the best performance in improving global cognitive function. Global cognition relies on the synergistic integration and efficient communication of multiple distributed brain networks. Multi-component exercise (typically combining aerobic, resistance, balance, and flexibility training) may exert its benefits through a “multiple pathway synergy” mechanism. Its aerobic component enhances cardiovascular function and cerebral blood flow perfusion (42, 43), optimizing the brain’s metabolic and nutritional environment; resistance training effectively promotes circulating levels of key neurotrophic factors such as insulin-like growth factor 1 and brain-derived neurotrophic factor (BDNF), directly supporting neuronal survival, synaptic plasticity, and neurogenesis (20, 44); while balance and coordination training further stimulates cerebellar and sensorimotor cortical networks (45). This simultaneous multi-dimensional stimulation of vascular, neurochemical, and neural networks gives it a unique advantage in enhancing comprehensive global cognitive performance.

This study found that AE could improve both executive and memory functions, suggesting its benefits for cognition are broad and foundational. Executive functions are primarily mediated by the prefrontal cortex. AE can significantly improve cardiorespiratory fitness, increase cerebral blood flow (particularly perfusion in the prefrontal regions), and provide the brain with a more stable energy supply (46, 47). Concurrently, AE is an effective stimulus for inducing BDNF release (47), which is crucial for the plasticity and functional connectivity of prefrontal neurons. For sedentary populations, regular AE may, through these mechanisms, systematically improve the efficiency and resilience of prefrontal networks, thereby establishing a physiological foundation for executive functions such as working memory, inhibitory control, and cognitive flexibility. The pronounced benefit of AE for memory function is closely related to the high plasticity of the hippocampus. Substantial evidence indicates that regular AE effectively promotes hippocampal neurogenesis (48), increases hippocampal volume (49), and enhances its functional connectivity with cortical networks (50). This is primarily achieved through increased hippocampal blood flow, upregulation of BDNF expression, and improved glucose metabolism (51). Therefore, for memory encoding and consolidation processes that primarily rely on the hippocampus and related medial temporal lobe structures, AE provides the most direct and efficient stimulus. The modifying effect of age differed across cognitive domains. Subgroup analysis using the median age of the included studies (68 years) as the cut-off showed that age significantly modified the effect of exercise on executive function (*p* for interaction = 0.014). Participants younger than 68 years exhibited a moderate and statistically significant improvement (SMD = 0.50), whereas those aged 68 years or older showed no benefit (SMD = 0.07). In contrast, for global cognition and memory, no significant age-dependent effect was observed. This age-specific effect may reflect reduced neuroplasticity or ceiling effects in older adults, and warrants further investigation.

However, these rankings should be interpreted with considerable caution, as the GRADE assessment indicated that most indirect and mixed comparisons underlying these network estimates were rated as very low certainty, primarily due to study limitations, imprecision, and publication bias concerns.

### 4.2 Comparison and integration with existing literature

The findings of this study both align with and offer new insights compared to prior literature. Firstly, the research reconfirms the broad benefits of exercise for cognitive function in sedentary populations (52). More importantly, through the quantitative ranking provided by the network meta-analysis, we move beyond the question of “whether exercise is effective” to reveal a finer-grained map of “which type of exercise is more effective for which cognitive function.” The dual advantage of AE in both memory and executive functions highlights its foundational role in cognitive health promotion, consistent with a large body of basic and clinical research focusing on hippocampal and prefrontal cortex functions (48–50). The improvement effect of ME on global cognitive function aligns with the findings of some previously published studies (53–55). Its advantage may stem from the integration of aerobic, resistance, balance, and other forms of training, which simultaneously promotes cerebral blood flow perfusion, releases neurotrophic factors, and enhances coordination across multiple brain networks. This comprehensive approach optimizes the vascular, metabolic, and neural plasticity foundations of the brain. The results of this study suggest that future development of cognitive enhancement programs should pay greater attention to the specific matching of exercise type with the target cognitive domain.

### 4.3 Strengths and limitations

The strength of this study lies in its pioneering application of network meta-analysis to quantitatively compare and rank the relative effects of multiple exercise intervention modalities across different cognitive domains in sedentary adults. This approach provides more informative, probability-based evidence, helping to transcend the limitations of traditional pairwise comparisons. The research process strictly adhered to the PRISMA-NMA reporting guidelines and systematically evaluated the risk of bias in the included studies as well as the overall quality of the evidence body, thereby enhancing methodological transparency and the reliability of the results.

However, this study also has several limitations. First, the number of included randomized controlled trials is limited, which may affect the precision of the related effect estimates and the stability of the rankings. Second, there is inherent heterogeneity among the primary studies regarding the specific parameters of the exercise interventions (e.g., intensity, frequency, duration), the assessment tools for cognitive function, and the characteristics of the participant populations (e.g., age range, baseline health status). Despite employing appropriate statistical models, residual heterogeneity may still influence the results. Furthermore, funnel plot analysis suggests the potential presence of publication bias or small-study effects. Importantly, GRADE assessment indicated that while direct comparisons provided moderate certainty evidence, the majority of indirect and mixed comparisons—which form the basis for many network estimates—were rated as very low confidence. This substantially tempers the strength of our comparative effectiveness claims and highlights the need for future direct comparison trials. Finally, In the present analysis, age subgroup analysis was performed by dividing participants into two groups using the median age of the included studies (68 years) as the cut-off. Due to the limited number of studies, further stratification into finer age bands (e.g., young, middle-aged, and early-senior adults) was not feasible. Consequently, the broad younger subgroup (21–68 years) encompasses a wide range of life stages, and potential differences in exercise efficacy across these stages could not be directly examined. These aspects warrant further investigation in future research.

### 4.4 Implications for practice and future research

Based on the findings of this study, the following principles can be considered when developing exercise prescriptions aimed at improving cognitive health in sedentary adults: AE should be recommended as a foundational and core component for enhancing overall cognitive health due to its significant benefits for both executive and memory functions. If resources and conditions permit, and the primary goal is to comprehensively improve global cognitive function, multi-component integrated training programs may be prioritized. It must always be emphasized that any form of regular physical activity is superior to sedentary behavior.

Future research should focus on the following areas: First, there is a need for more large-scale, high-quality, long-term follow-up randomized controlled trials, particularly those that directly compare the effects of key modalities such as AE, RE, and ME. Second, efforts should be made to promote the standardization of cognitive assessment tools and the reporting of exercise intervention protocols to enhance comparability across studies and the efficiency of evidence synthesis. Third, it is essential to further explore the dose–response relationships of exercise interventions and to examine the moderating effects of individual difference factors—such as age, genetic background, and baseline health status—on outcomes. Fourth, combining multimodal neuroimaging and molecular biomarker technologies will help clarify the biological pathways through which different exercise modes produce specific cognitive benefits. Finally, long-term follow-up studies are needed to investigate the sustainability of cognitive benefits derived from exercise and their potential role in preventing cognitive impairment.

## 5 Conclusion

This systematic review and network meta-analysis suggest that exercise interventions may improve cognitive function in sedentary adults, with different exercise modalities showing potential domain-specific effects. ME exhibited the greatest potential for enhancing global cognitive function, while AE demonstrated potential advantages for executive function and memory. However, the very low certainty of most indirect comparisons in the network underscores the need for cautious interpretation. These findings provide hypothesis-generating evidence for developing individualized exercise prescriptions, but confirmatory high-quality direct comparison trials are urgently needed.
