# Supplementary material for: Effects of exercise interventions on cognitive function in sedentary adults: a systematic review and network meta-analysis
Source: Front Public Health. 2026 May 14;14:1794345. doi: 10.3389/fpubh.2026.1794345 (PMC13217309; doi:10.3389/fpubh.2026.1794345)
Supplement: Supplementary file 1 [file Data_Sheet_1.docx]

**Supplemental files**

**Contents**

[**Supplementary file 1: Search strategy** 1](#_Toc229082478)

[1.1 Search strategy for Pubmed 1](#_Toc229082479)

[1.2 Search strategy for Web of Science 2](#_Toc229082480)

[1.3 Search strategy for EBSCO 3](#_Toc229082481)

[1.4 Search strategy for Scopus 4](#_Toc229082482)

[1.5 Search strategy for Embase 4](#_Toc229082483)

[1.6 Search strategy for Cochrane Library 5](#_Toc229082484)

[**Supplementary file 2: The measurement tools for different outcomes** 7](#_Toc229082485)

[**Supplementary file 3: Demographic characteristics of included·studies** 8](#_Toc229082486)

[**Supplementary file 4: Results of the risk-of-bias assessment using the Cochrane Risk of Bias 2.0 tool** 10](#_Toc229082487)

[**Supplementary file 5: Pairwise analysis** 11](#_Toc229082488)

[**Supplementary file 6: Network meta-analysis** 16](#_Toc229082489)

[**Supplementary File 7: GRADE assessment of the certainty of evidence** 19](#_Toc229082490)

[**Supplementary File 8: Abbreviation list** 21](#_Toc229082491)

[**Included studies** 22](#_Toc229082492)

**Supplementary file 1: Search strategy**

1.1 Search strategy for Pubmed

(((((((((((((Sedentary Behavior[MeSH Terms]) OR (Behavior, Sedentary[Title/Abstract])) OR (Sedentary Behaviors[Title/Abstract])) OR (Sedentary Lifestyle[Title/Abstract])) OR (Lifestyle, Sedentary[Title/Abstract])) OR (Physical Inactivity[Title/Abstract])) OR (Inactivity, Physical[Title/Abstract])) OR (Lack of Physical Activity[Title/Abstract])) OR (Sedentary Time[Title/Abstract])) OR (Sedentary Times[Title/Abstract])) OR (Time, Sedentary[Title/Abstract])) AND ((((((((((((((((((((((((((((((((((((((((((((Exercise[MeSH Terms]) OR (Exercises[Title/Abstract])) OR (Exercise, Physical[Title/Abstract])) OR (Exercises, Physical[Title/Abstract])) OR (Physical Exercise[Title/Abstract])) OR (Physical Exercises[Title/Abstract])) OR (Exercise, Aerobic[Title/Abstract])) OR (Aerobic Exercise[Title/Abstract])) OR (Aerobic Exercises[Title/Abstract])) OR (Exercises, Aerobic[Title/Abstract])) OR (Exercise, Isometric[Title/Abstract])) OR (Exercises, Isometric[Title/Abstract])) OR (Isometric Exercises[Title/Abstract])) OR (Isometric Exercise[Title/Abstract])) OR (Acute Exercise[Title/Abstract])) OR (Acute Exercises[Title/Abstract])) OR (Exercise, Acute[Title/Abstract])) OR (Exercises, Acute[Title/Abstract])) OR (Exercise Training[Title/Abstract])) OR (Exercise Trainings[Title/Abstract])) OR (Training, Exercise[Title/Abstract])) OR (Trainings, Exercise[Title/Abstract])) OR (Physical Activity[Title/Abstract])) OR (Activities, Physical[Title/Abstract])) OR (Activity, Physical[Title/Abstract])) OR (Physical Activities[Title/Abstract])) OR (sport*[Title/Abstract])) OR (walk*[Title/Abstract])) OR (swim*[Title/Abstract])) OR (yoga[Title/Abstract])) OR (qigong[Title/Abstract])) OR (baduanjing[Title/Abstract])) OR (pilate[Title/Abstract])) OR (taichi[Title/Abstract])) OR (resistance[Title/Abstract])) OR (training[Title/Abstract])) OR (Exercise Training[Title/Abstract])) OR (Exercise Trainings[Title/Abstract])) OR (moderate intensity continuous training[Title/Abstract])) OR (resistance training[Title/Abstract])) OR (strength training[Title/Abstract])) OR (combines training[Title/Abstract])) OR (sprint interval training[Title/Abstract])) OR (high intensity interval training[Title/Abstract]))) AND ((((((((Cognition[MeSH Terms]) OR (Cognitions[Title/Abstract])) OR (Cognitive Function[Title/Abstract])) OR (Cognitive Functions[Title/Abstract])) OR (Function, Cognitive[Title/Abstract])) OR (Functions, Cognitive[Title/Abstract])) OR (Insight[Title/Abstract])) OR (Insights[Title/Abstract]))) AND (((randomized controlled trial[Title/Abstract]) OR (randomized[Title/Abstract])) OR (placebo[Title/Abstract]))

1.2 Search strategy for Web of Science

((TS=("Sedentary Behavior" OR "Behavior, Sedentary" OR "Sedentary Behaviors" OR "Sedentary Lifestyle" OR "Lifestyle, Sedentary" OR "Physical Inactivity" OR "Inactivity, Physical" OR "Lack of Physical Activity" OR "Sedentary Time" OR "Sedentary Times" OR "Time, Sedentary")) And (TS=("Exercise" OR "Exercises" OR "Exercise, Physical" OR "Exercises, Physical" OR "Physical Exercise" OR "Physical Exercises" OR "Exercise, Aerobic" OR "Aerobic Exercise" OR "Aerobic Exercises" OR "Exercises, Aerobic" OR "Exercise, Isometric" OR "Exercises, Isometric" OR "Isometric Exercises" OR "Isometric Exercise" OR "Acute Exercise" OR "Acute Exercises" OR "Exercise, Acute" OR "Exercises, Acute" OR "Exercise Training" OR "Exercise Trainings" OR "Training, Exercise" OR "Trainings, Exercise" OR "Physical Activity" OR "Activities, Physical" OR "Activity, Physical" OR "Physical Activities" OR "sport*" OR "walk*" OR "swim*" OR "yoga" OR "qigong" OR "baduanjing" OR "pilate" OR "taichi" OR "resistance" OR "training" OR "Exercise Training" OR "Exercise Trainings" OR "moderate intensity continuous training" OR "resistance training" OR "strength training" OR "combines training" OR "sprint interval training" OR "high intensity interval training")) And (TS=（"Cognition" OR "Cognitions" OR "Cognitive Function" OR "Cognitive Functions" OR "Function, Cognitive" OR "Functions, Cognitive" OR "Insight" OR "Insights"）) And (TS=("randomized controlled trial" OR "controlled clinical trial" OR "Randomized Controlled Trials as Topic" OR "Clinical Trials as Topic" OR "Controlled Clinical Trials as Topic" OR "Random Allocation" OR "randomized" OR "randomised" OR "randomization" OR "randomly allocated" OR "RCT")))

1.3 Search strategy for EBSCO

((AB "Sedentary Behavior" OR AB "Behavior, Sedentary" OR AB "Sedentary Behaviors" OR AB "Sedentary Lifestyle" OR AB "Lifestyle, Sedentary" OR AB "Physical Inactivity" OR AB "Inactivity, Physical" OR AB "Lack of Physical Activity" OR AB "Sedentary Time" OR AB "Sedentary Times" OR AB "Time, Sedentary") AND (AB "Exercise" OR AB "Exercises" OR AB "Exercise, Physical" OR AB "Exercises, Physical" OR AB "Physical Exercise" OR AB "Physical Exercises" OR AB "Exercise, Aerobic" OR AB "Aerobic Exercise" OR AB "Aerobic Exercises" OR AB "Exercises, Aerobic" OR AB "Exercise, Isometric" OR AB "Exercises, Isometric" OR AB "Isometric Exercises" OR AB "Isometric Exercise" OR AB "Acute Exercise" OR AB "Acute Exercises" OR AB "Exercise, Acute" OR AB "Exercises, Acute" OR AB "Exercise Training" OR AB "Exercise Trainings" OR AB "Training, Exercise" OR AB "Trainings, Exercise" OR AB "Physical Activity" OR AB "Activities, Physical" OR AB "Activity, Physical" OR AB "Physical Activities" OR AB "sport*" OR AB "walk*" OR AB "swim*" OR AB "yoga" OR AB "qigong" OR AB "baduanjing" OR AB "pilate" OR AB "taichi" OR AB "resistance" OR AB "training" OR AB "Exercise Training" OR AB "Exercise Trainings" OR AB "moderate intensity continuous training" OR AB "resistance training" OR AB "strength training" OR AB "combines training" OR AB "sprint interval training" OR AB "high intensity interval training") AND (AB "Cognition" OR AB "Cognitions" OR AB "Cognitive Function" OR AB "Cognitive Functions" OR AB "Function, Cognitive" OR AB "Functions, Cognitive" OR AB "Insight" OR AB "Insights") AND (AB "randomized controlled trial" OR AB "controlled clinical trial" OR AB "Randomized Controlled Trials as Topic" OR AB "Clinical Trials as Topic" OR AB "Controlled Clinical Trials as Topic" OR AB "Random Allocation" OR AB "randomized" OR AB "randomised" OR AB "randomization" OR AB "randomly allocated" OR AB "RCT" OR AB "clinical trial" OR AB "clinical stud*"))

1.4 Search strategy for Scopus

((TITLE-ABS-KEY："Sedentary Behavior" OR "Behavior, Sedentary" OR "Sedentary Behaviors" OR "Sedentary Lifestyle" OR "Lifestyle, Sedentary" OR "Physical Inactivity" OR "Inactivity, Physical" OR "Lack of Physical Activity" OR "Sedentary Time" OR "Sedentary Times" OR "Time, Sedentary") And (TITLE-ABS-KEY："Exercise" OR "Exercises" OR "Exercise, Physical" OR "Exercises, Physical" OR "Physical Exercise" OR "Physical Exercises" OR "Exercise, Aerobic" OR "Aerobic Exercise" OR "Aerobic Exercises" OR "Exercises, Aerobic" OR "Exercise, Isometric" OR "Exercises, Isometric" OR "Isometric Exercises" OR "Isometric Exercise" OR "Acute Exercise" OR "Acute Exercises" OR "Exercise, Acute" OR "Exercises, Acute" OR "Exercise Training" OR "Exercise Trainings" OR "Training, Exercise" OR "Trainings, Exercise" OR "Physical Activity" OR "Activities, Physical" OR "Activity, Physical" OR "Physical Activities" OR "sport*" OR "walk*" OR "swim*" OR "yoga" OR "qigong" OR "baduanjing" OR "pilate" OR "taichi" OR "resistance" OR "training" OR "Exercise Training" OR "Exercise Trainings" OR "moderate intensity continuous training" OR "resistance training" OR "strength training" OR "combines training" OR "sprint interval training" OR "high intensity interval training") And (TITLE-ABS-KEY："Cognition" OR "Cognitions" OR "Cognitive Function" OR "Cognitive Functions" OR "Function, Cognitive" OR "Functions, Cognitive" OR "Insight" OR "Insights") And (TITLE-ABS-KEY："randomized controlled trial" OR "controlled clinical trial" OR "Randomized Controlled Trials as Topic" OR "Clinical Trials as Topic" OR "Controlled Clinical Trials as Topic" OR "Random Allocation" OR "randomized" OR "randomised" OR "randomization" OR "randomly allocated" OR "RCT"))

1.5 Search strategy for Embase

((('sedentary lifestyle'/exp) OR ('behavior, sedentary':ab,ti OR 'sedentary behaviors':ab,ti OR 'sedentary behavior':ab,ti OR 'lifestyle, sedentary':ab,ti OR 'physical inactivity':ab,ti OR 'inactivity, physical':ab,ti OR 'lack of physical activity':ab,ti OR 'sedentary time':ab,ti OR 'sedentary times':ab,ti OR 'time, sedentary':ab,ti)) AND (('exercise'/exp) OR ('exercises':ab,ti OR 'exercise, physical':ab,ti OR 'exercises, physical':ab,ti OR 'physical exercise':ab,ti OR 'physical exercises':ab,ti OR 'exercise, aerobic':ab,ti OR 'aerobic exercise':ab,ti OR 'aerobic exercises':ab,ti OR 'exercises, aerobic':ab,ti OR 'exercise, isometric':ab,ti OR 'exercises, isometric':ab,ti OR 'isometric exercises':ab,ti OR 'isometric exercise':ab,ti OR 'acute exercise':ab,ti OR 'acute exercises':ab,ti OR 'exercise, acute':ab,ti OR 'exercises, acute':ab,ti OR 'exercise training':ab,ti OR 'exercise trainings':ab,ti OR 'training, exercise':ab,ti OR 'trainings, exercise':ab,ti OR 'physical activity':ab,ti OR 'activities, physical':ab,ti OR 'activity, physical':ab,ti OR 'physical activities':ab,ti OR 'sport*':ab,ti OR 'walk*':ab,ti OR 'swim*':ab,ti OR 'yoga':ab,ti OR 'qigong':ab,ti OR 'baduanjing':ab,ti OR 'pilate':ab,ti OR 'taichi':ab,ti OR 'resistance':ab,ti OR 'training':ab,ti OR 'exercise training':ab,ti OR 'exercise trainings':ab,ti OR 'moderate intensity continuous training':ab,ti OR 'resistance training':ab,ti OR 'strength training':ab,ti OR 'combines training':ab,ti OR 'sprint interval training':ab,ti OR 'high intensity interval training':ab,ti)) AND (('cognition'/exp) OR ('cognitions':ab,ti OR 'cognitive function':ab,ti OR 'cognitive functions':ab,ti OR 'function, cognitive':ab,ti OR 'functions, cognitive':ab,ti OR 'insight':ab,ti OR 'insights':ab,ti)) AND (('randomized controlled trial'/exp) OR ('controlled clinical trial':ab,ti OR 'randomized controlled trials as topic':ab,ti OR 'clinical trials as topic':ab,ti OR 'controlled clinical trials as topic':ab,ti OR 'random allocation':ab,ti OR 'randomized':ab,ti OR 'randomised':ab,ti OR 'randomization':ab,ti OR 'randomly allocated':ab,ti OR 'rct':ab,ti OR 'clinical trial':ab,ti OR 'clinical stud*':ab,ti)))

1.6 Search strategy for Cochrane Library

(((MeSH descriptor: [Sedentary Behavior] explode all trees) OR ((Behavior, Sedentary):ti,ab,kw OR (Sedentary Behaviors):ti,ab,kw OR (Sedentary Lifestyle):ti,ab,kw OR (Lifestyle, Sedentary):ti,ab,kw OR (Physical Inactivity):ti,ab,kw OR (Inactivity, Physical):ti,ab,kw OR (Lack of Physical Activity):ti,ab,kw OR (Sedentary Time):ti,ab,kw OR (Sedentary Times):ti,ab,kw OR (Time, Sedentary):ti,ab,kw)) AND ((MeSH descriptor: [Exercise] explode all trees) OR ((Exercises):ti,ab,kw OR (Exercise, Physical):ti,ab,kw OR (Exercises, Physical):ti,ab,kw OR (Physical Exercise):ti,ab,kw OR (Physical Exercises):ti,ab,kw OR (Exercise, Aerobic):ti,ab,kw OR (Aerobic Exercise):ti,ab,kw OR (Aerobic Exercises):ti,ab,kw OR (Exercises, Aerobic):ti,ab,kw OR (Exercise, Isometric):ti,ab,kw OR (Exercises, Isometric):ti,ab,kw OR (Isometric Exercises):ti,ab,kw OR (Isometric Exercise):ti,ab,kw OR (Acute Exercise):ti,ab,kw OR (Acute Exercises):ti,ab,kw OR (Exercise, Acute):ti,ab,kw OR (Exercises, Acute):ti,ab,kw OR (Exercise Training):ti,ab,kw OR (Exercise Trainings):ti,ab,kw OR (Training, Exercise):ti,ab,kw OR (Trainings, Exercise):ti,ab,kw OR (Physical Activity):ti,ab,kw OR (Activities, Physical):ti,ab,kw OR (Activity, Physical):ti,ab,kw OR (Physical Activities):ti,ab,kw OR (sport*):ti,ab,kw OR (walk*):ti,ab,kw OR (swim*):ti,ab,kw OR (yoga):ti,ab,kw OR (qigong):ti,ab,kw OR (baduanjing):ti,ab,kw OR (pilate):ti,ab,kw OR (taichi):ti,ab,kw OR (resistance):ti,ab,kw OR (training):ti,ab,kw OR (Exercise Training):ti,ab,kw OR (Exercise Trainings):ti,ab,kw OR (moderate intensity continuous training):ti,ab,kw OR (resistance training):ti,ab,kw OR (strength training):ti,ab,kw OR (combines training):ti,ab,kw OR (sprint interval training):ti,ab,kw OR (high intensity interval training):ti,ab,kw)) AND ((MeSH descriptor: [Cognition] explode all trees) OR ((Cognitions):ti,ab,kw OR (Cognitive Function):ti,ab,kw OR (Cognitive Functions):ti,ab,kw OR (Function, Cognitive):ti,ab,kw OR (Functions, Cognitive):ti,ab,kw OR (Insight):ti,ab,kw OR (Insights):ti,ab,kw)))

**Supplementary file 2: The measurement tools for different outcomes**

Table 1 The measurement tools for different outcomes

| Primary outcomes | |
| --- | --- |
| Global cognition | The Montreal cognitive assessment (MoCA); Flanker test; National  Institute of Health (NIH) Toolbox; Event-related brain potentials (ERP) test; Mini Mental State Examination (MMSE); Z-scores of Attention, Neuropsychological Test Battery; Verbal fluency, and Episodic memory for Non-demented Adults (ZAVEN); The Symptom Impact Inventory |
| Executive cognition | The Stroop Task; Wisconsin Card Sorting Test (WCST); Halstead-Reitan Neuropsychological Test Battery (verbal fluency); Picture Arrangement. (WAIS-III; Wechsler Adult Intelligence Scale, 3rd edition); Flanker test; National Institute of Health (NIH) Toolbox; Event-related brain potentials (ERP) test; “Trails B-Trails A” (switching); Neuropsychological Test Battery; California Verbal Learning Test-II (CVLT); F-A-S Verbal Fluency Test (FAS]); Digit Symbol Coding (DSC) task |
| Memory function | Free Word Recall; Rey-Osterrieth Complex Figure Test (ROCF); National Institute of Health (NIH) Toolbox; California Verbal Learning Test Second Edition (CVLT-II); Neuropsychological Test Battery; Color-Word Interference (CWI); The German equivalent of the Auditory Verbal Learning Test; International Shopping List Test (ISLT); Hopkins Verbal Learning Test (HVLT-R) |

**Supplementary file 3: Demographic characteristics of included·studies**

This supplementary file shows the Demographic characteristics of included studies. The study indicates the name of the author and the year of' publication. The exact dose parameter indicates the exact estimated·METs per week that participants accumulated in the study. The frequency is the number of davs those participants were involved in physical activity. USA, the United states of America. HIIT, High-Intensity Interval Training. MICT, Moderate-Intensity Continuous Training.

| **Study** | **Country** | **N** | **Gender** | **Mean Age**  **(SD/Range)** | **Interventions** | **Intervention duration**  **(weeks)** | **Frequency** | **Time/session** | **Exact dose** | **Outcomes measured** |
| --- | --- | --- | --- | --- | --- | --- | --- | --- | --- | --- |
| Cedric T 2016 | France | 36 | Femals:26  Males:10 | 60-75 | Swimming | 21 | 2 | 60 | 660 | Global cognitive function: Stroop task |
| Cedric T 2010 | France | 24 | Femals:13  Males:11 | 70.7±4.2 | Aerobic exercise | 12 | 3 | 60 | 900 | Executive function: WCST |
| Juliana 2015 | Brazil | 69 | Femals:47  Males:22 | 82.4 ± 2.4 | Resistance exercise  Multicomponent exercise | 16  16 | 3  3 | 60  60 | 1080  821.7 | Global cognitive function: MoCA  Executive function: verbal fluency |
| H.K. Antunes 2015 | Brazil | 46 | Males:46 | 66.97 ± 4.8 | Aerobic exercise | 24 | 3 | 20-60 | 1224 | Executive function: WAIS-III  Memory function: Free Word Recall |
| Hanna 2015 | Brazil | 59 | Femals:34  Males:25 | 60-75 | Aerobic exercise | 24 | 3 | 20-60 | 1224 | Executive function: WCST  Memory function: ROCF |
| Guilherme 2024 | Canada | 23 | - | 55-65 | Multicomponent exercise | 3 | 7 | 60-72 | 3003 | Global cognitive function: Flanker test  Executive function: Flanker test |
| Edward 2022 | Australia | 27 | Femals:21  Males:6 | 66.52 ± 7.88 | Aerobic exercise | 16 | 2-4 | 40-45 | 507 | Global cognitive function: NIH Toolbox  Executive function: NIH Toolbox  Memory function: NIH Toolbox |
| E. ÇETİN 2010 | Turkey | 23 | - | 70.33 ± 8.69 | Walking | 24 | 3 | 50 | 570 | Global cognitive function: ERP test  Executive function: ERP test |
| Sandra 2013 | USA | 37 | Femals:27  Males:10 | 64.0 ± 3.9 | Aerobic exercise | 12 | 3 | 60 | 735 | Executive function: Trails B-Trails A  Memory function: CVLT-II |
| Julian 2019 | USA | 23 | - | 64.86 ± 4.66 | Treadmill walking | 26 | 3 | 50 | 1050 | Global cognitive function: MMSE  Executive function: CVLT  Memory function: CWI |
| Kirsten 2011 | Germany | 54 | Femals:34  Males:20 | 40-56 | Cycling | 24 | 2 | 60 | 816 | Executive function: Stroop task  Memory function: The German equivalent of the Auditory Verbal Learning Test |
| HUANG 2024 | China | 63 | Femals:33  Males:30 | 21.62 ± 1.75 | Resistance exercise  HIIT  MICT | 8 | 3 | 30  25  40 | 315  660  630 | Executive function: Stroop task |
| Jonasson 2017 | Sweden | 58 | Femals:32  Males:26 | 68.68 ± 2.72 | Aerobic exercise | 24 | 3 | 30-60 | 985.5 | Global cognitive function: Neuropsychological Test Battery  Executive function: Neuropsychological Test Battery  Memory function: Neuropsychological Test Battery |
| Chandra 2019 | Brazil | 52 | Femals:40  Males:12 | 72.25 ± 7.78 | Multicomponent exercise | 24 | 2 | 60 | 409.5 | Global cognitive function: MMSE |
| Elena 2021 | USA | 17 | Femals:13  Males:4 | 70.1 ± 6.8 | Aerobic exercise | 12 | 3 | 40 | 456 | Global cognitive function: Verbal fluency, and Episodic memory for Non-demented Adults (ZAVEN)  Executive function: FAS  Memory function: ISLT |
| Francisco 2016 | Spain | 100 | Femals:54  Males:46 | 80.0 ± 3.6 | Multicomponent exercise | 24 | 5 | 65 | 942 | Global cognitive function: MMSE |
| Kaycee 2015 | USA | 1476 | - | 70-89 | Multicomponent exercise | 48 | 5-6 | 50 | 1035 | Executive function: DSC  Memory function: HVLT-R |

**Supplementary file 4: Results of the risk-of-bias assessment using the Cochrane Risk of Bias 2.0 tool**

| **Study** | **bias arising from the randomization proces** | **bias due to deviations from intended interventions** | **bias due to missing outcome data** | **bias in measurement of the outcome** | **bias in selection of the reported result** | **Risk category** |
| --- | --- | --- | --- | --- | --- | --- |
| Cedric T 2016 | Some concerns | LOW risk | LOW risk | LOW risk | LOW risk | Some concerns |
| Cedric T 2010 | Some concerns | LOW risk | LOW risk | LOW risk | LOW risk | Some concerns |
| Juliana 2015 | LOW risk | LOW risk | LOW risk | LOW risk | LOW risk | LOW risk |
| H.K. Antunes 2015 | Some concerns | LOW risk | LOW risk | Some concerns | LOW risk | Some concerns |
| Hanna 2015 | Some concerns | LOW risk | LOW risk | LOW risk | LOW risk | Some concerns |
| Guilherme 2024 | Some concerns | LOW risk | LOW risk | LOW risk | LOW risk | Some concerns |
| Edward 2022 | Some concerns | LOW risk | LOW risk | LOW risk | LOW risk | Some concerns |
| E. ÇETİN 2010 | Some concerns | LOW risk | High risk | LOW risk | LOW risk | High risk |
| Sandra 2013 | Some concerns | LOW risk | LOW risk | LOW risk | LOW risk | Some concerns |
| Julian 2019 | Some concerns | LOW risk | LOW risk | LOW risk | LOW risk | Some concerns |
| Kirsten 2011 | Some concerns | LOW risk | High risk | LOW risk | LOW risk | High risk |
| HUANG 2024 | Some concerns | LOW risk | LOW risk | LOW risk | LOW risk | Some concerns |
| Jonasson 2017 | High risk | LOW risk | LOW risk | LOW risk | LOW risk | High risk |
| Chandra 2019 | Some concerns | LOW risk | LOW risk | LOW risk | LOW risk | Some concerns |
| Elena 2021 | LOW risk | LOW risk | LOW risk | LOW risk | LOW risk | LOW risk |
| Francisco 2016 | LOW risk | LOW risk | LOW risk | LOW risk | LOW risk | LOW risk |
| Kaycee 2015 | Some concerns | LOW risk | LOW risk | LOW risk | LOW risk | Some concerns |

**Supplementary file 5: Pairwise analysis**


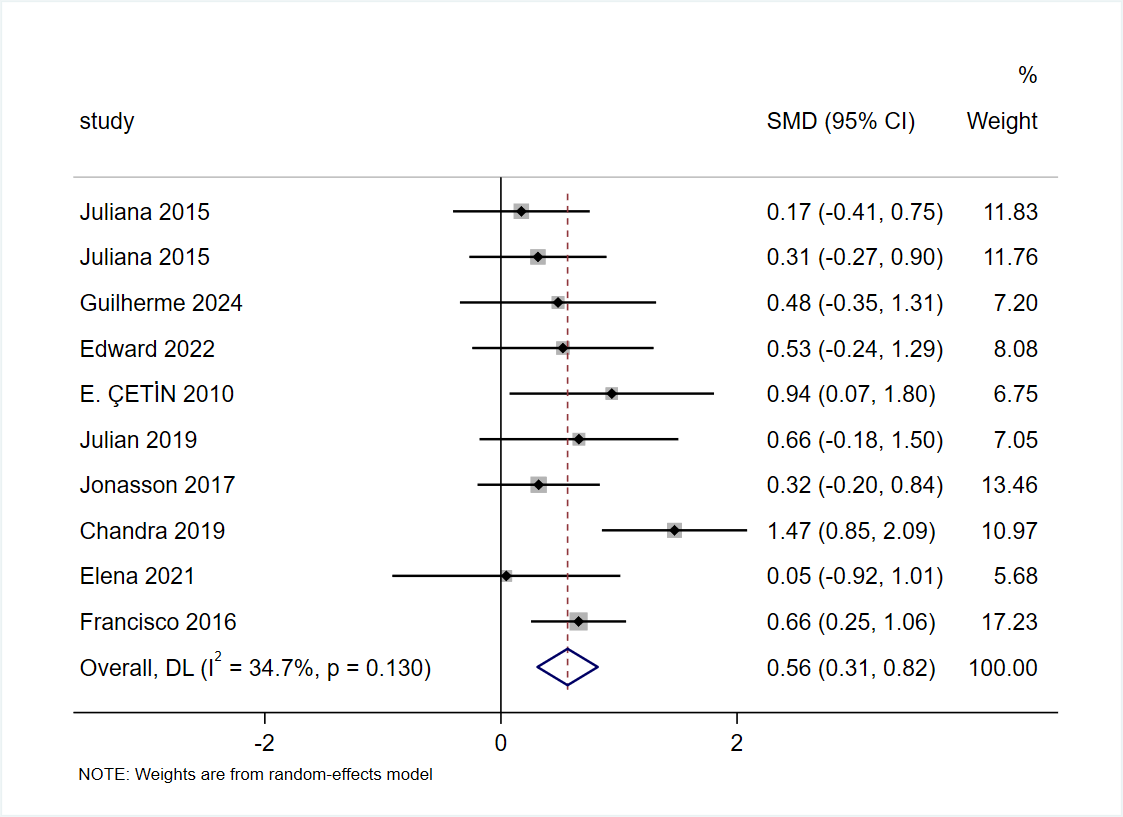


Figure 1. Forest plot of pairwise meta-analysis for global cognitive function


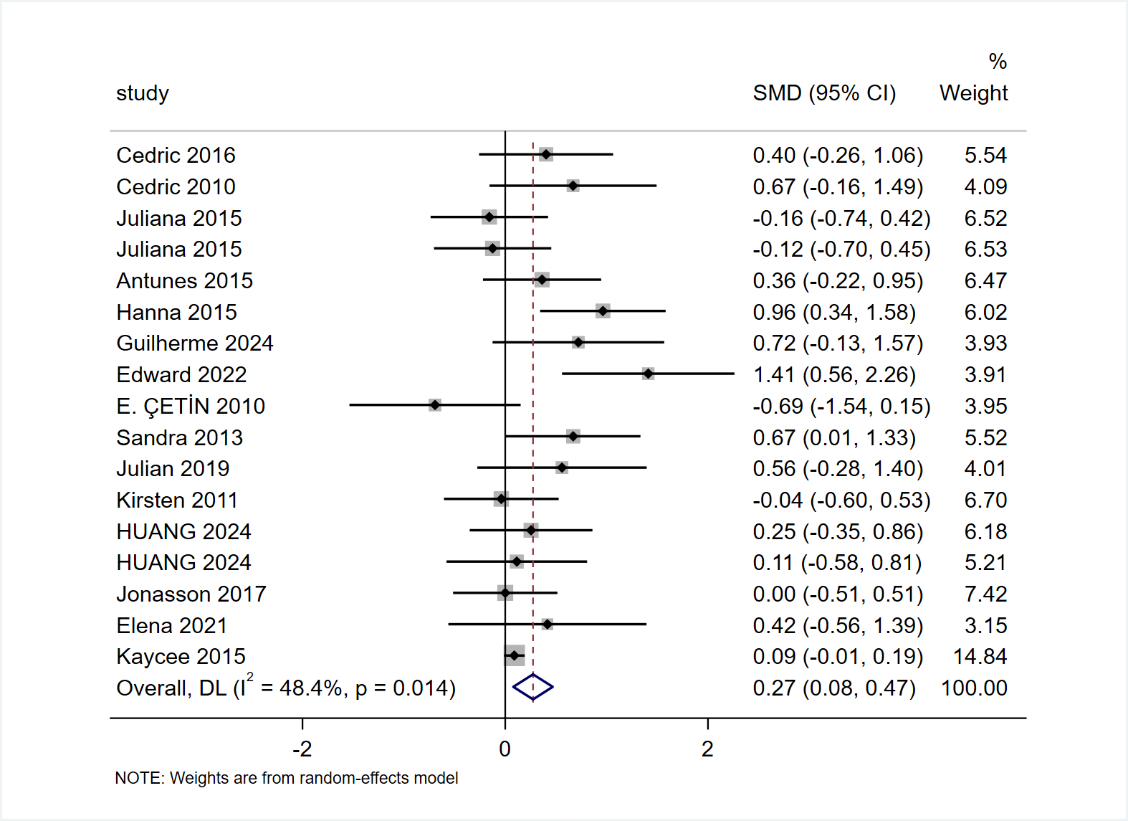


Figure 2. Forest plot of pairwise meta-analysis for executive function.


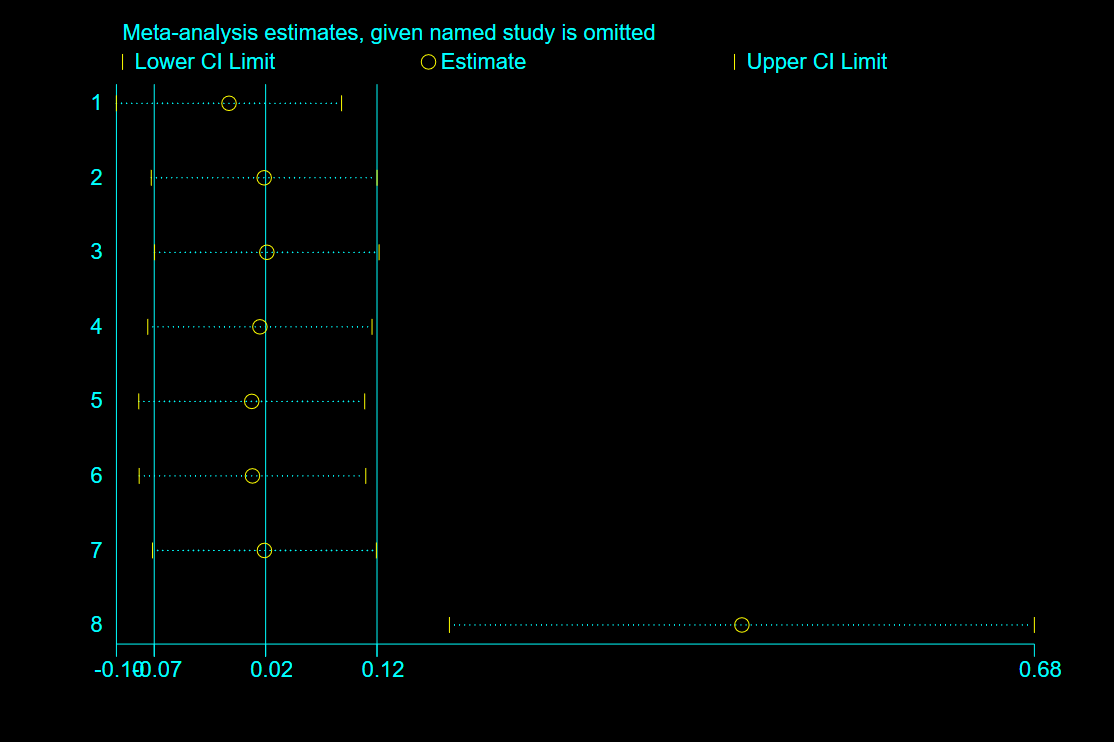


Figure 3. Sensitivity analysis for memory function.


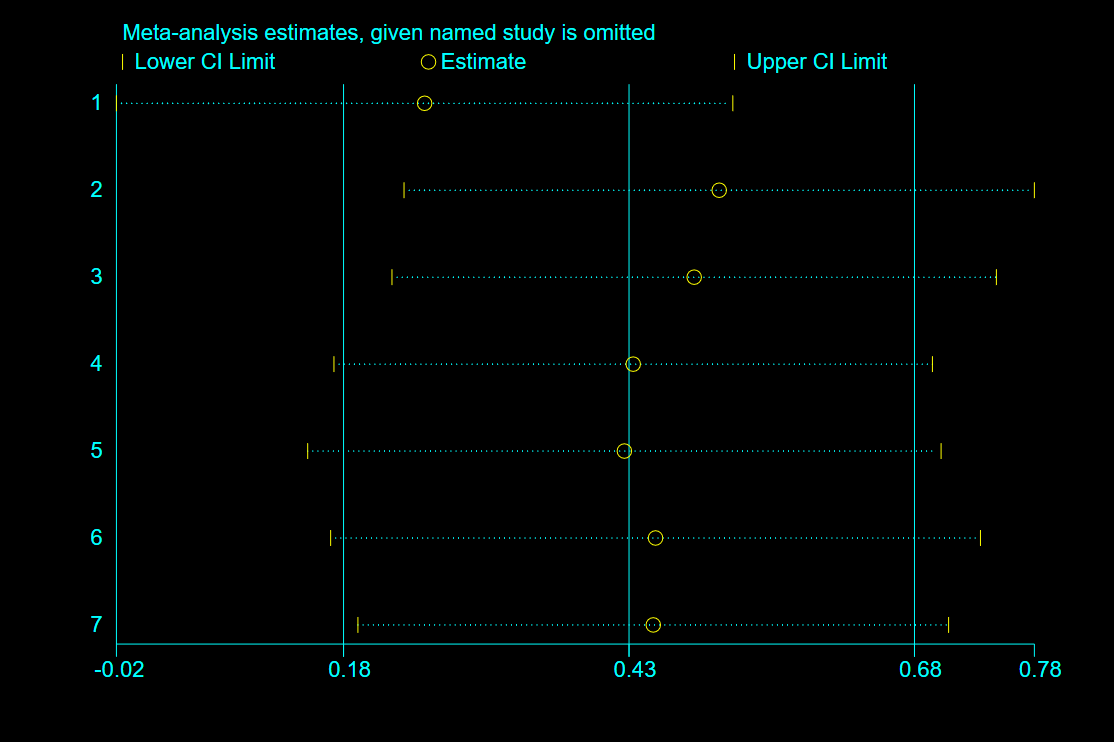


Figure 4. Sensitivity analysis for memory function after excluding the LIFE trial.


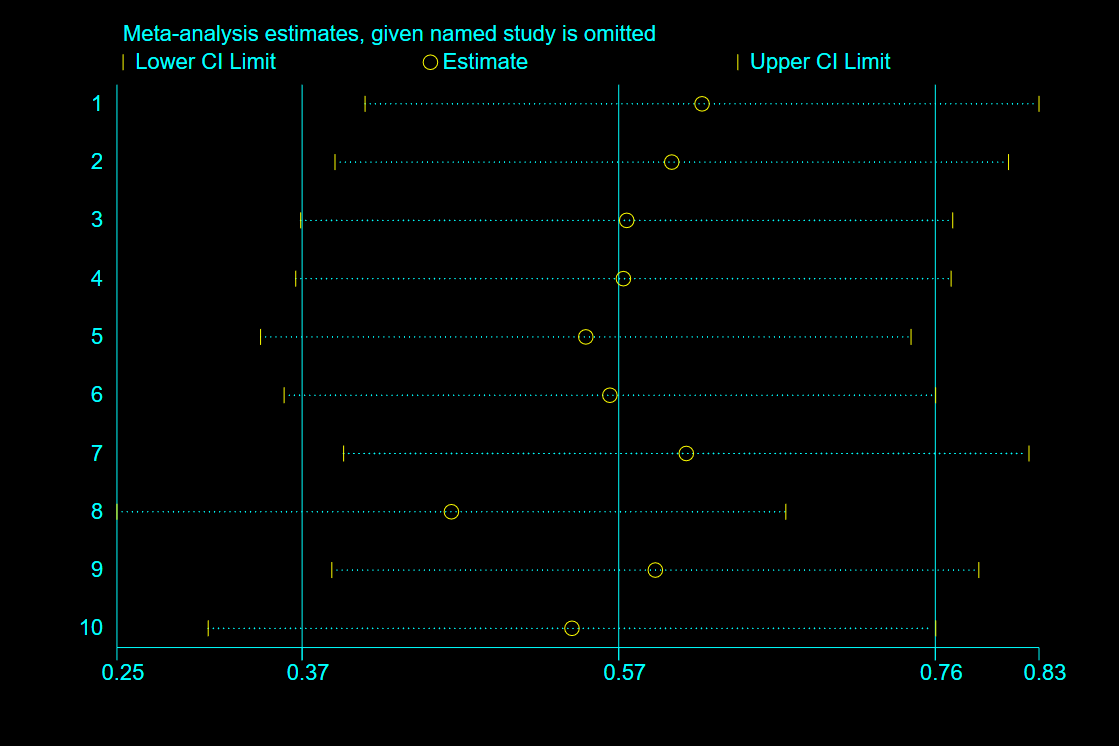


Figure 5. Sensitivity analysis for global cognitive function.


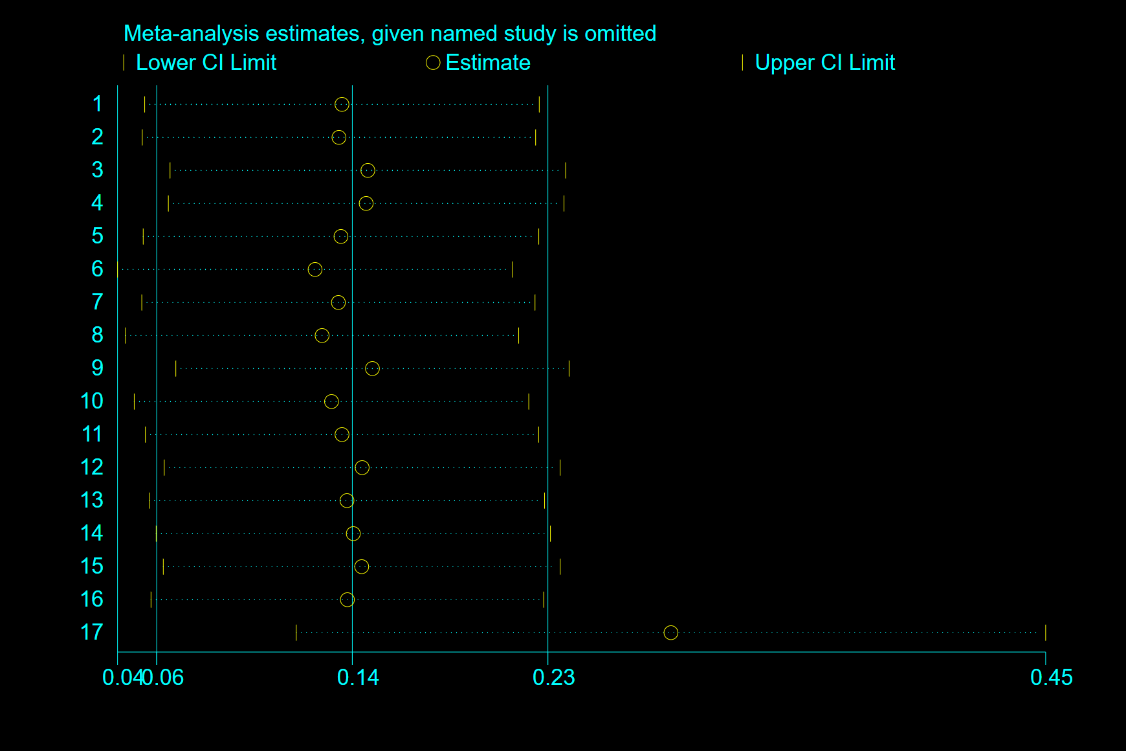


Figure 6. Sensitivity analysis for executive function.


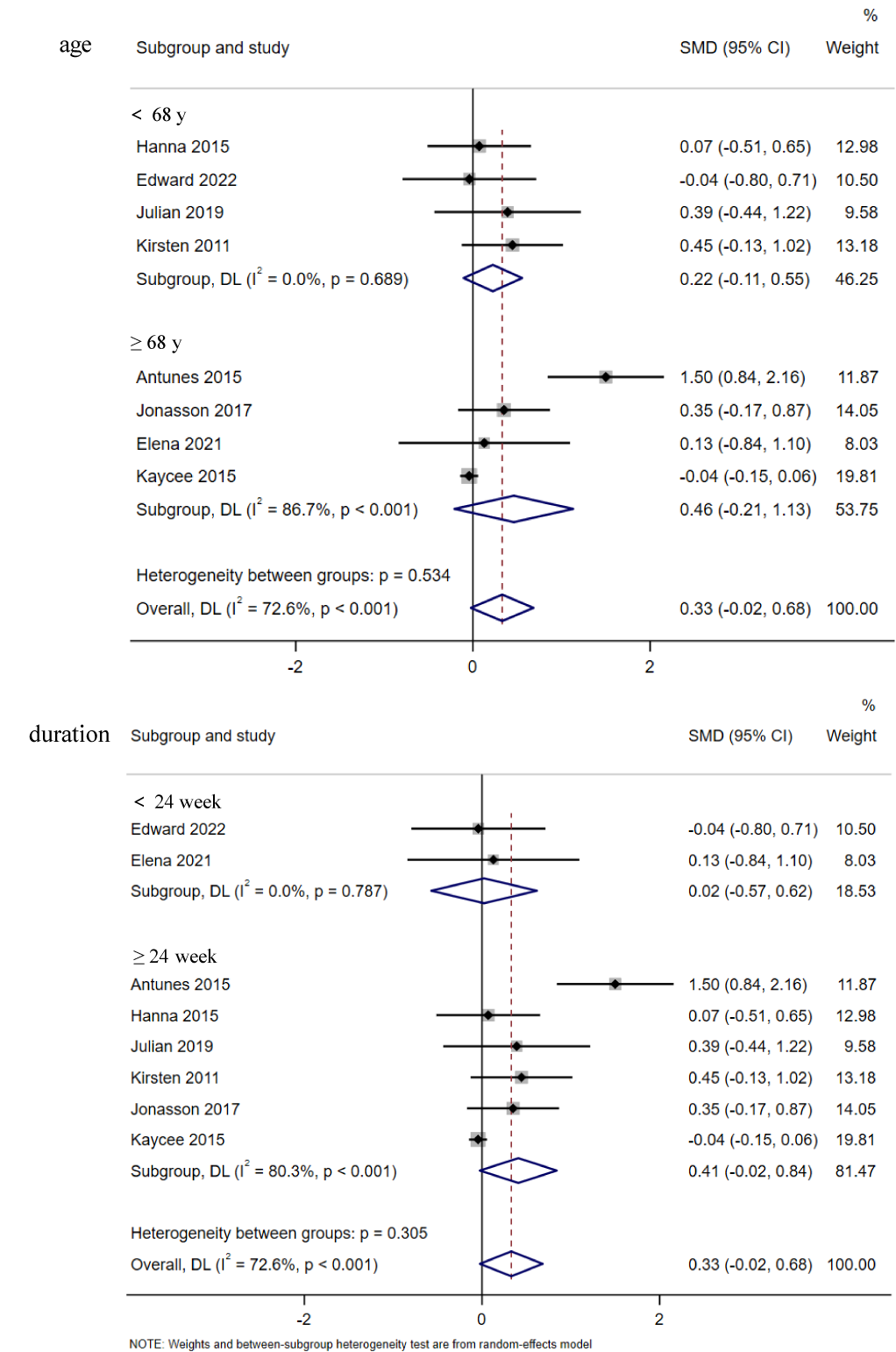


Figure 7. Subgroup analysis for memory function.


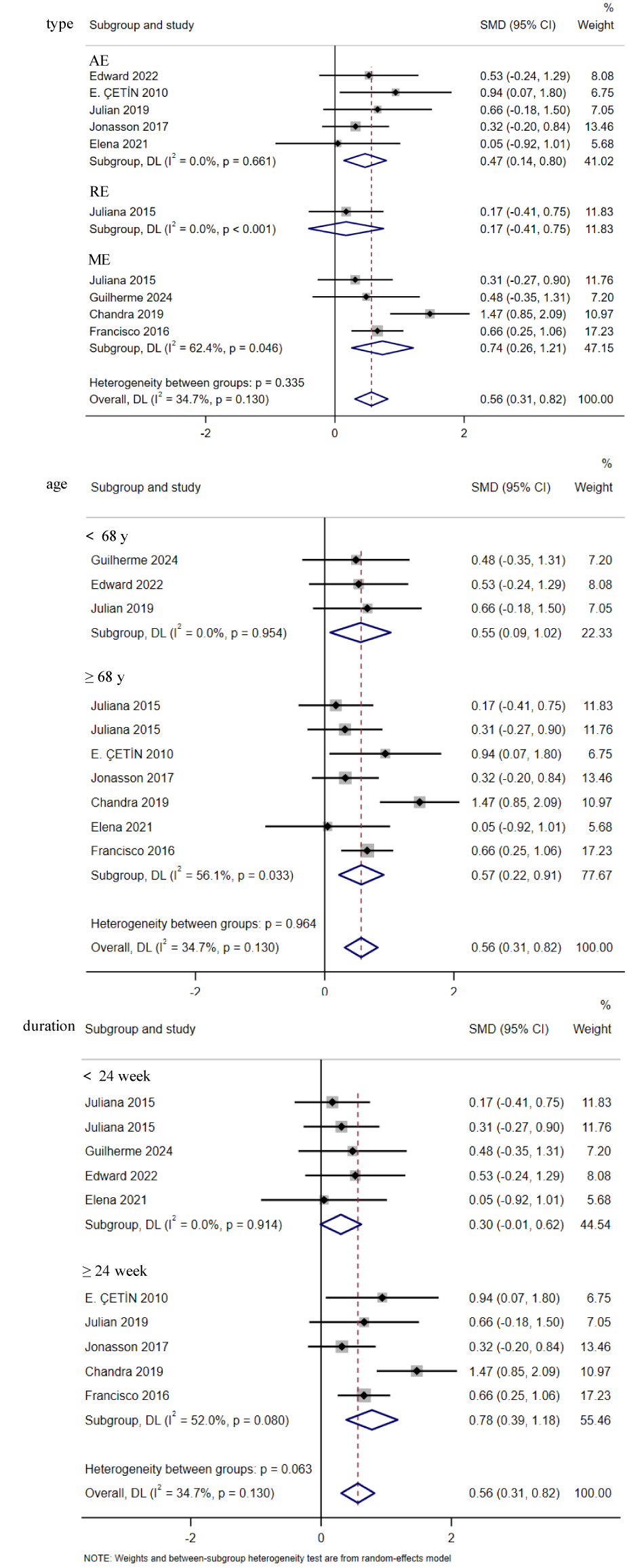


Figure 8. Subgroup analysis for global cognitive function. AE : aerobic exercise; RE: resistance exercise; ME: multicomponent exercise.


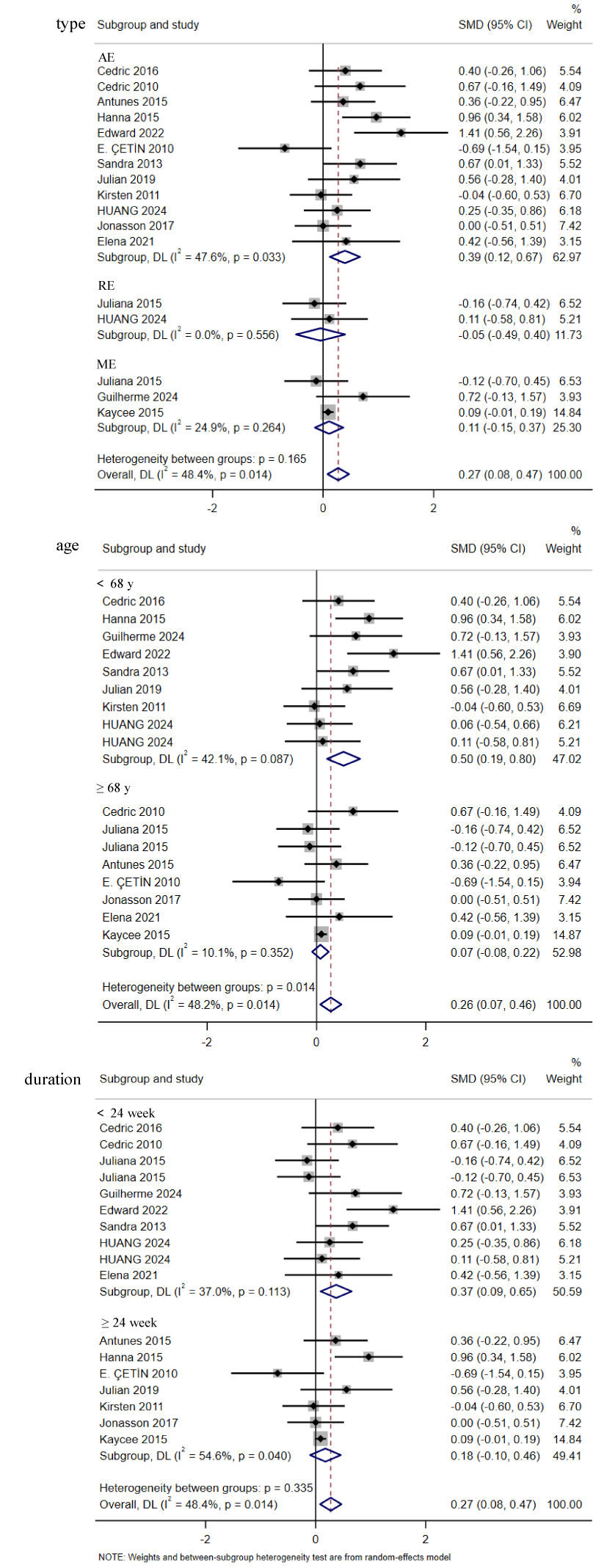


Figure 9. Subgroup analysis for executive function. AE : aerobic exercise; RE: resistance exercise; ME: multicomponent exercise.

**Supplementary file 6: Network meta-analysis**


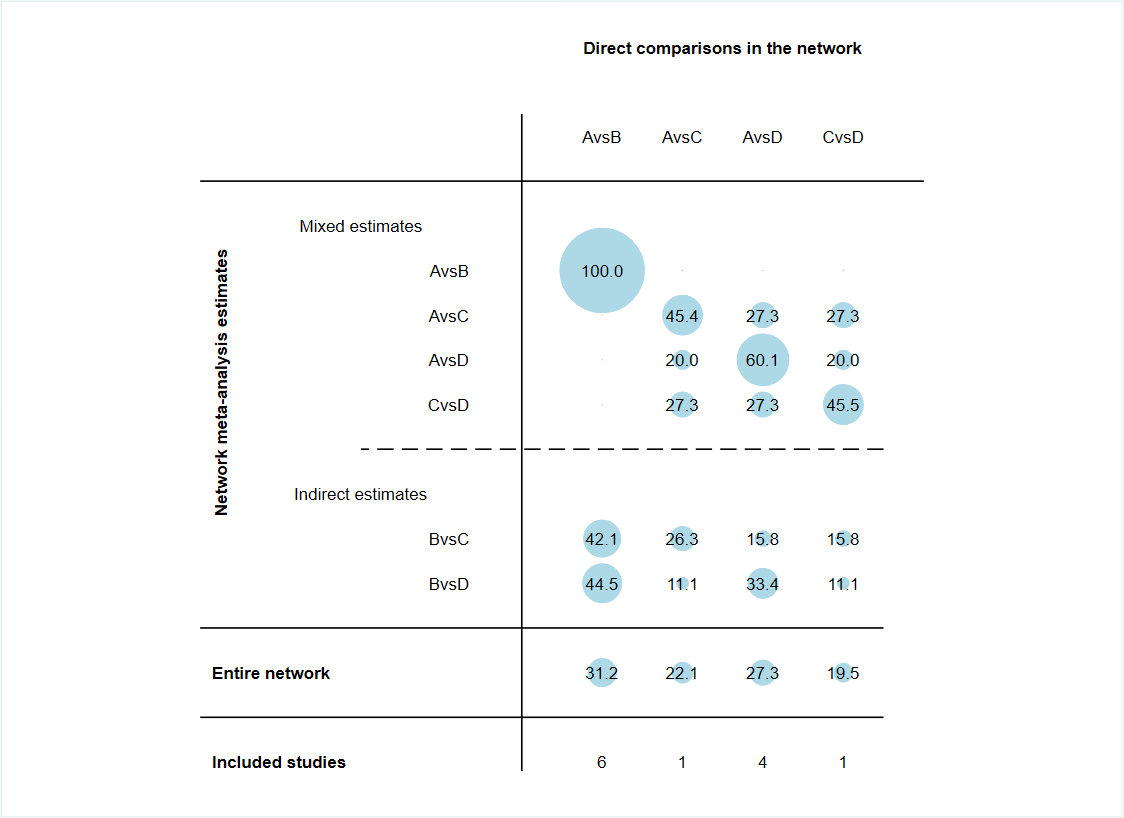


Figure 10. Network contribution plot for global cognitive function. A: control group; B: aerobic exercise; C: resistance exercise; D: multicomponent exercise.


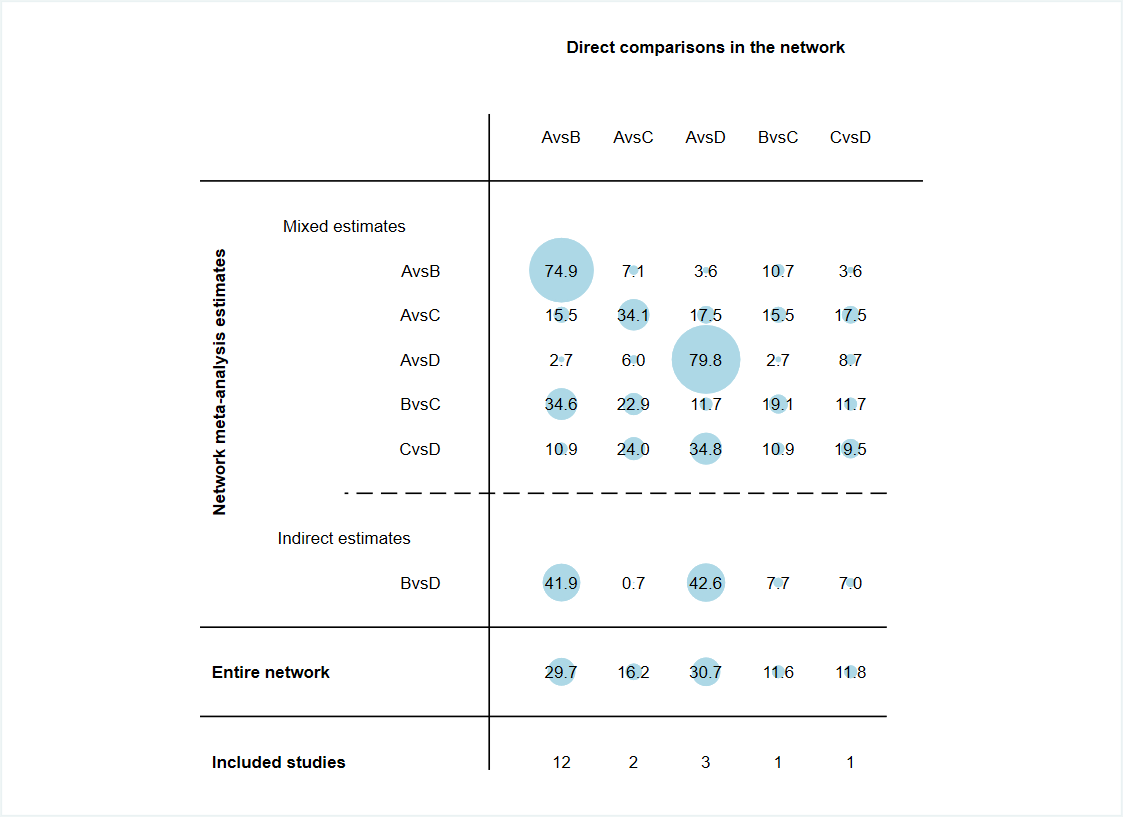


Figure 11. Network contribution plot for executive function. A: control group; B: aerobic exercise; C: resistance exercise; D: multicomponent exercise.

Table 2 Evaluation of inconsistency using loop-specific heterogeneity estimates for global cognitive function

| Loop | IF | seIF | z_value | p_value | CI_95 | Loop_Heterog_tau2 |
| --- | --- | --- | --- | --- | --- | --- |
| CON-RE-ME | 0.527 | 0.447 | 1.179 | 0.238 | (0.00,1.40) | 0.000 |

*Note.* CON: control group; RE: resistance exercise; ME: multicomponent exercise.

Table 3 Evaluation of inconsistency using loop-specific heterogeneity estimates for executive function

| Loop | IF | seIF | z_value | p_value | CI_95 | Loop_Heterog_tau2 |
| --- | --- | --- | --- | --- | --- | --- |
| CON-AE-RE | 0.307 | 0.578 | 0.531 | 0.596 | (0.00,1.44) | 0.1 |
| CON-RE-ME | 0.093 | 0.376 | 0.247 | 0.805 | (0.00,0.83) | 0 |

*Note.* CON: control group; AE : aerobic exercise; RE: resistance exercise; ME: multicomponent exercise.

Table 4. Evaluation of inconsistency using fitting inconsistency models

|  | Global cognitive function | Executive function |
| --- | --- | --- |
| chi2 | 1.77 | 0.75 |
| Prob > chi2 | 0.1833 | 0.8614 |

Table 5. Evaluation of inconsistency using node-splitting for global cognitive function

| Side | Direct | | Indirect | | Difference | | |
| --- | --- | --- | --- | --- | --- | --- | --- |
|  | Coef. | Std. Err. | Coef. | Std. Err. | Coef. | Std. Err. | P>z |
| A C* | 0.1738838 | 0.3594201 | 1.280439 | 0.7491291 | -1.106556 | 0.8315971 | 0.183 |
| C D* | 0.1304129 | 0.3592769 | 1.236969 | 0.7493352 | -1.106556 | 0.8315971 | 0.183 |

*Note.* A: control group; C: resistance exercise; D: multicomponent exercise.

Table 6. Evaluation of inconsistency using node-splitting for executive function

| Side | Direct | | Indirect | | Difference | | |
| --- | --- | --- | --- | --- | --- | --- | --- |
|  | Coef. | Std. Err. | Coef. | Std. Err. | Coef. | Std. Err. | P>z |
| A B* | 0.3800754 | 0.1271441 | -0.1166329 | 1.025267 | 0.4967083 | 1.035463 | 0.631 |
| A C* | -0.0313547 | 0.2952413 | 0.4582815 | 0.5569966 | -0.4896362 | 0.6296854 | 0.437 |
| A D* | 0.131745 | 0.2047776 | 0.6284534 | 1.015071 | -0.4967083 | 1.035463 | 0.631 |
| B C | -0.1219277 | 0.4019244 | -0.4280804 | 0.3566477 | 0.3061527 | 0.5285766 | 0.562 |
| C D | 0.0469366 | 0.4059833 | 0.1111303 | 0.4502799 | -0.0641937 | 0.6062543 | 0.916 |

*Note.* A: control group; B: aerobic exercise; C: resistance exercise; D: multicomponent exercise.

Table 7. League table for global cognitive function

| AE | -0.09 (-0.87,0.69) | 0.25 (-0.29,0.80) | -0.47 (-0.87,-0.07) |
| --- | --- | --- | --- |
| 0.09 (-0.69,0.87) | RE | 0.34 (-0.33,1.01) | -0.38 (-1.05,0.29) |
| -0.25 (-0.80,0.29) | -0.34 (-1.01,0.33) | ME | -0.72 (-1.09,-0.35) |
| 0.47 (0.07,0.87) | 0.38 (-0.29,1.05) | 0.72 (0.35,1.09) | CON |

*Note.* CON: control group; AE: aerobic exercise; RE: resistance exercise; ME: multicomponent exercise.

Table 8. League table for executive function

| AE | -0.29 (-0.80,0.21) | -0.23 (-0.65,0.20) | -0.37 (-0.61,-0.13) |
| --- | --- | --- | --- |
| 0.29 (-0.21,0.80) | RE | 0.07 (-0.47,0.61) | -0.07 (-0.56,0.41) |
| 0.23 (-0.20,0.65) | -0.07 (-0.61,0.47) | ME | -0.14 (-0.51,0.22) |
| 0.37 (0.13,0.61) | 0.07 (-0.41,0.56) | 0.14 (-0.22,0.51) | CON |

*Note.* CON: control group; AE: aerobic exercise; RE: resistance exercise; ME: multicomponent exercise.


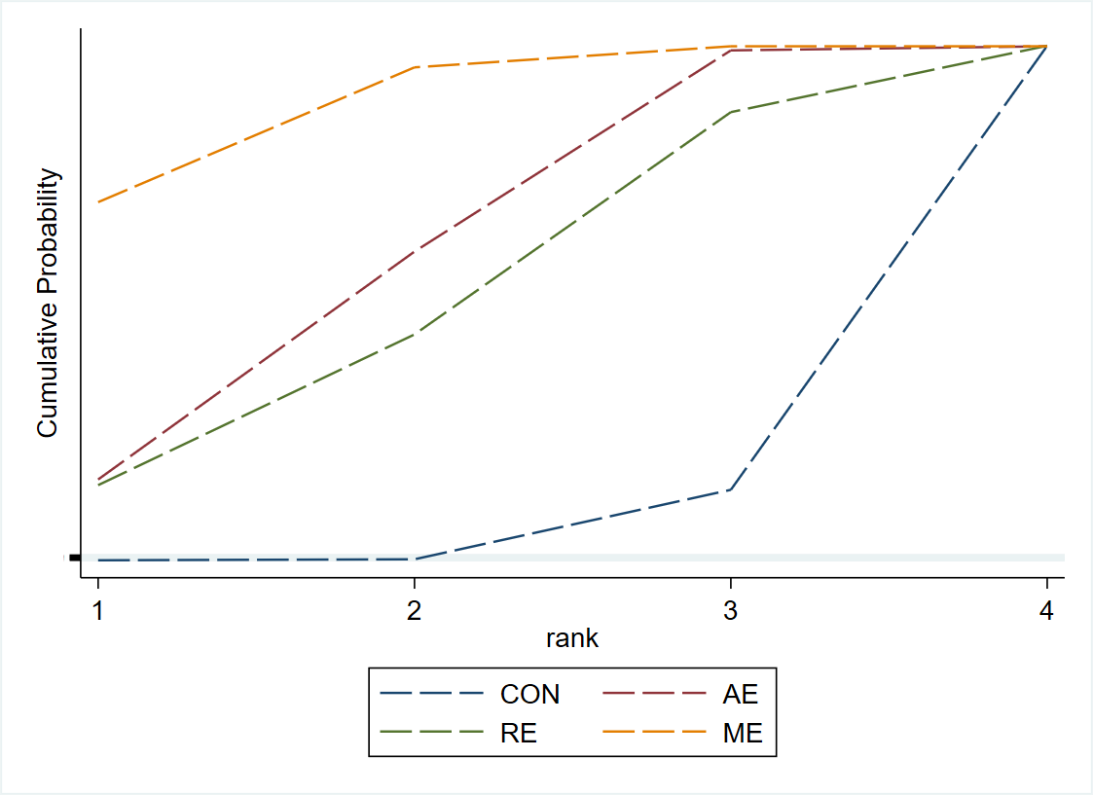


Figure 12. Ranking Probabilities for global cognitive function (SUCRA Values). CON: control group; AE: aerobic exercise; RE: resistance exercise; ME: multicomponent exercise.


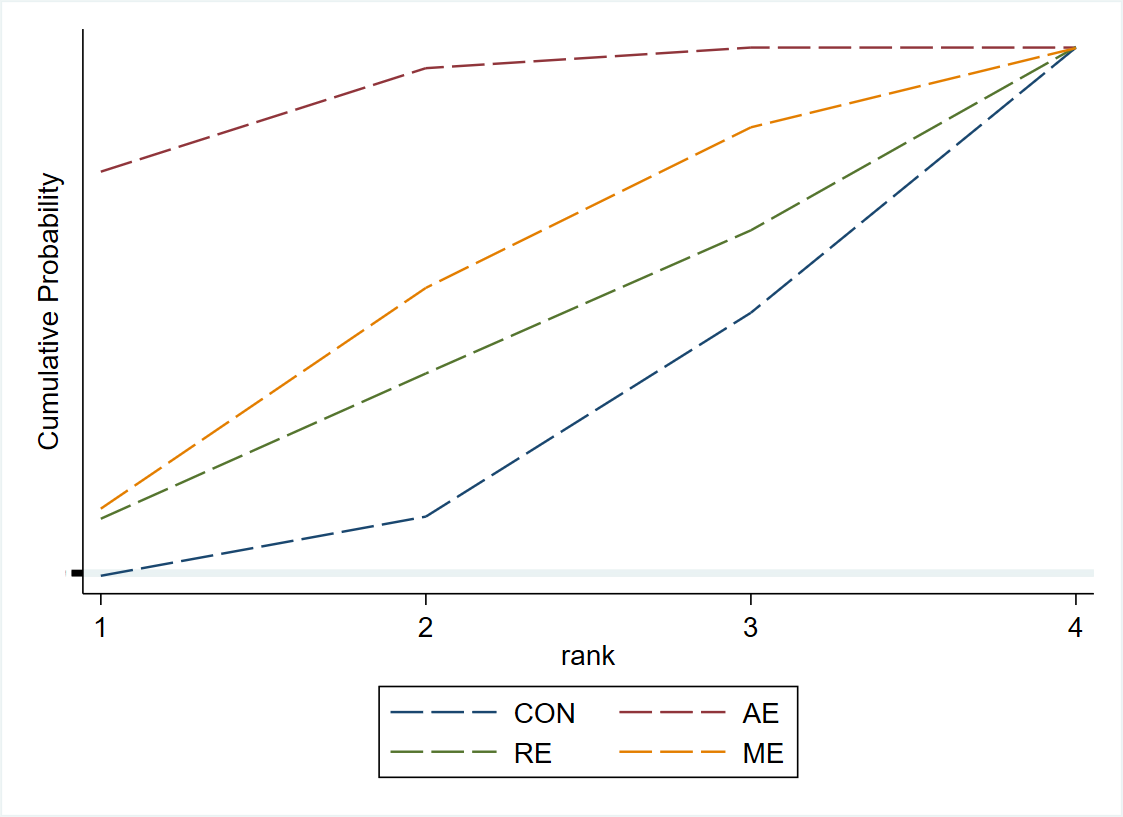


Figure 13. Ranking Probabilities for executive function (SUCRA Values). CON: control group; AE: aerobic exercise; RE: resistance exercise; ME: multicomponent exercise.

Table 9. Ranking Probabilities (SUCRA Values) for global cognitive function

| Treatment | SUCRA | PrBest | MeanRank |
| --- | --- | --- | --- |
| CON | 4.6 | 0 | 3.9 |
| AE | 58.3 | 15.7 | 2.2 |
| RE | 48.5 | 14.6 | 2.5 |
| ME | 88.5 | 69.6 | 1.3 |

*Note.* CON: control group; AE: aerobic exercise; RE: resistance exercise; ME: multicomponent exercise.

Table 10. Ranking Probabilities (SUCRA Values) for executive function

| Treatment | SUCRA | PrBest | MeanRank |
| --- | --- | --- | --- |
| CON | 20.3 | 0 | 3.4 |
| AE | 90.8 | 76.5 | 1.3 |
| RE | 38.1 | 10.8 | 2.9 |
| ME | 50.7 | 12.7 | 2.5 |

*Note.* CON: control group; AE: aerobic exercise; RE: resistance exercise; ME: multicomponent exercise.

**Supplementary File 7: GRADE assessment of the certainty of evidence**


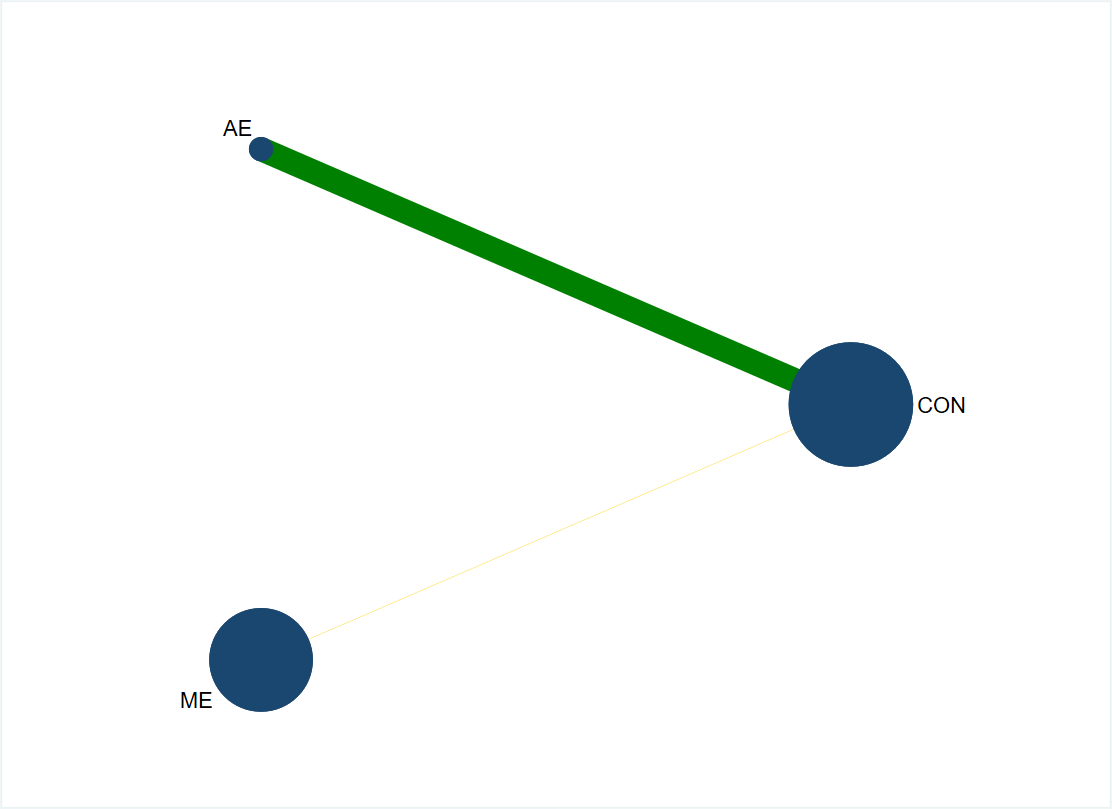


Figure 14. Network plot of the evidence for memory function. CON: control group; AE: aerobic exercise; ME: multicomponent exercise.


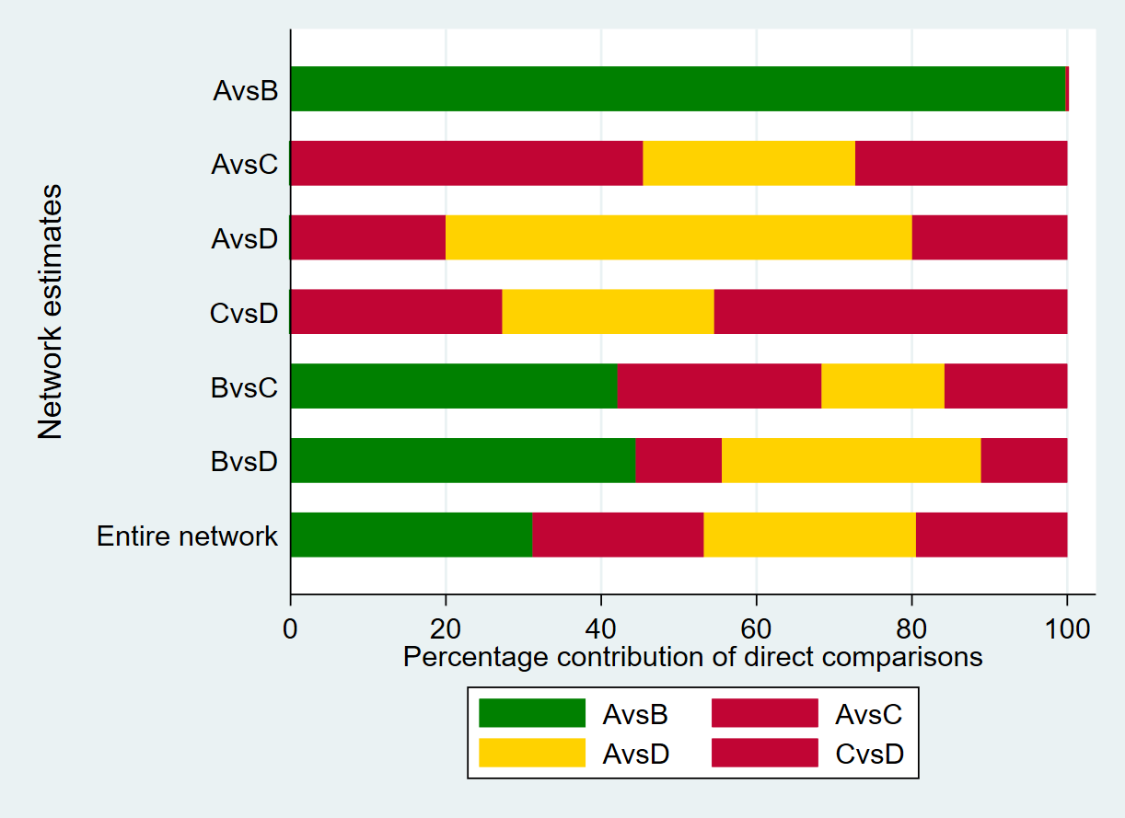


Figure 15. Bar plot of evidence contributions for global cognitive function. A: control group; B: aerobic exercise; C: resistance exercise; D: multicomponent exercise.


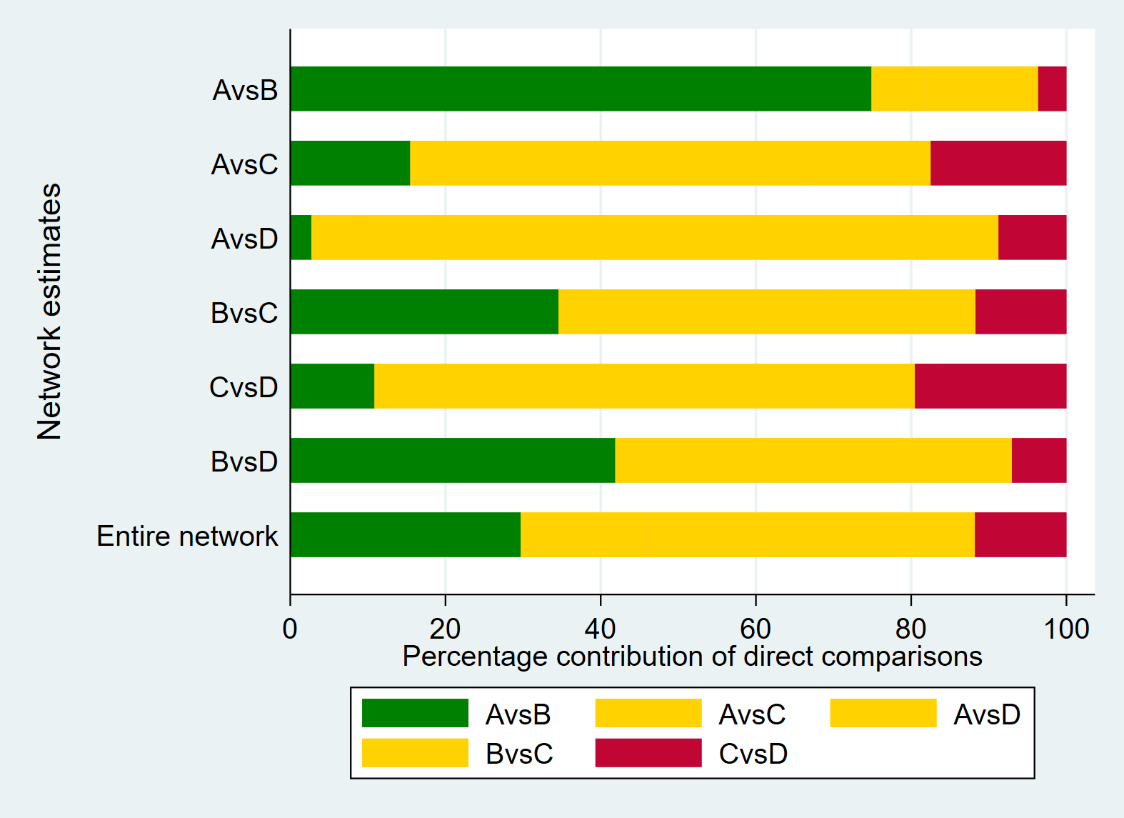


Figure 16. Bar plot of evidence contributions for executive function. A: control group; B: aerobic exercise; C: resistance exercise; D: multicomponent exercise.


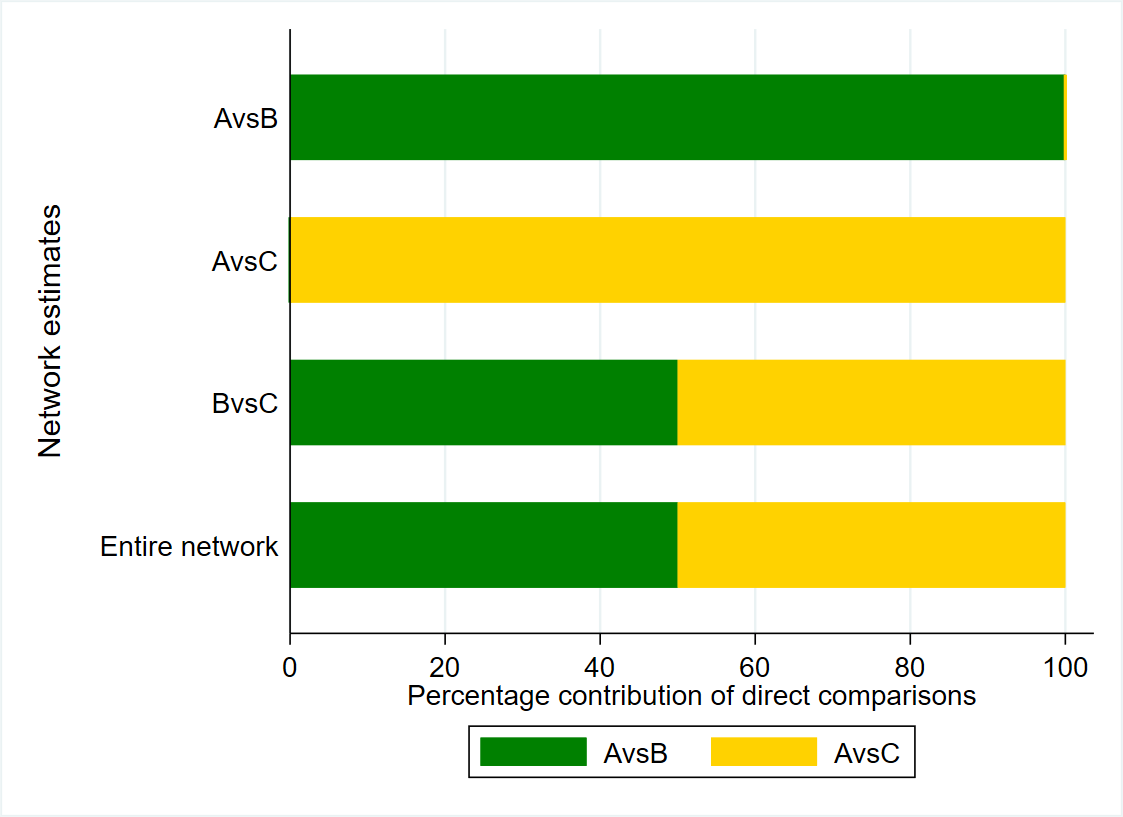


Figure 17. Bar plot of evidence contributions for memory function. A: control group; B: aerobic exercise; C: multicomponent exercise.

Table 11. Summary of our confidence in effect estimates and ranking of treatments for global cognitive function

| Comparison | Nature of the evidence | Confidence | Downgrading due to |
| --- | --- | --- | --- |
| A vs B | Direct | Moderate | Publication bias^1^ |
| A vs C | Mixed | Very LOW | Study limitations^2^, Publication bias^1^ |
| A vs D | Mixed | Very LOW | Study limitations^2^, Publication bias^1^ |
| C vs D | Mixed | Very LOW | Study limitations^2^, Publication bias^1^ |
| B vs C | Indirect | Very LOW | Study limitations^1^, Indirectness^1^, Publication bias^1^ |
| B vs D | Indirect | Very LOW | Study limitations^1^, Indirectness^1^, Publication bias^1^ |
| Ranking of treatments |  | Very LOW | Study limitations^2^, Publication bias^1^ |

*Note.* A: control group; B: aerobic exercise; C: resistance exercise; D: multicomponent exercise.

Table 12. Summary of our confidence in effect estimates and ranking of treatments for executive function

| Comparison | Nature of the evidence | Confidence | Downgrading due to |
| --- | --- | --- | --- |
| A vs B | Mixed | Moderate | Publication bias^1^ |
| A vs C | Mixed | Very LOW | Study limitations^2^, Publication bias^1^ |
| A vs D | Mixed | Very LOW | Study limitations^2^, Publication bias^1^ |
| C vs D | Mixed | Very LOW | Study limitations^2^, Publication bias^1^ |
| B vs C | Mixed | Very LOW | Study limitations^1^, Indirectness^1^, Publication bias^1^ |
| Ranking of treatments |  | LOW | Study limitations^1^, Publication bias^1^ |

*Note.* A: control group; B: aerobic exercise; C: resistance exercise; D: multicomponent exercise.

Table 13. Summary of our confidence in effect estimates for memory function

| Comparison | Nature of the evidence | Confidence | Downgrading due to |
| --- | --- | --- | --- |
| A vs B | Direct | Moderate | Imprecision^1^ |
| A vs C | Direct | LOW | Study limitations^1^, Imprecision^1^ |

*Note.* A: control group; B: aerobic exercise; C: multicomponent exercise.

**Supplementary File 8: Abbreviation list**

Table 14. Abbreviation list

| Full text | Abbreviation |
| --- | --- |
| aerobic exercise | AE |
| resistance exercise | RE |
| multicomponent exercise | ME |
| Preferred Reporting Items for Systematic Reviews and Meta-Analyses | PRISMA |
| Preferred Reporting Items for Systematic Reviews and Meta-Analyses for network meta-analyses | PRISMA-NMA |
| Risk of Bias 2.0 | RoB 2.0 |
| The Grading of Recommendations Assessment, Development and Evaluation | GRADE |
| standardized mean differences | SMDs |
| The surface under the cumulative ranking curve | SUCRA |
| control group | CON |

**Included studies**

1. Albinet CT, Abou-Dest A, André N, Audiffren M. Executive functions improvement following a 5-month aquaerobics program in older adults: Role of cardiac vagal control in inhibition performance. Biological Psychology. 2016;115:69-77.
2. Albinet CT, Boucard G, Bouquet CA, Audiffren M. Increased heart rate variability and executive performance after aerobic training in the elderly. European Journal of Applied Physiology. 2010;109(4):617-24.
3. Ansai JH, Rebelatto JR. Effect of two physical exercise protocols on cognition and depressive symptoms in oldest-old people: A randomized controlled trial. Geriatrics and Gerontology International. 2015;15(9):1127-34.
4. Antunes HK, Mello MT, Santos-Galduróz RF, Galduróz JCF, Aquino Lemos V, Tufik S, et al. Effects of a physical fitness program on memory and blood viscosity in sedentary elderly men. Brazilian Journal of Medical and Biological Research. 2015;48(9):805-12.
5. Antunes HKM, De Mello MT, De Aquino Lemos V, Santos-Galduróz RF, Galdieri LC, Bueno OFA, et al. Aerobic physical exercise improved the cognitive function of elderly males but did not modify their blood homocysteine levels. Dementia and Geriatric Cognitive Disorders Extra. 2015;5(1):13-24.
6. Balbim GM, Falck RS, Barha CK, Tai D, Best JR, Hajj-Boutros G, et al. Exercise counters the negative impact of bed rest on executive functions in middle-aged and older adults: A proof-of-concept randomized controlled trial. Maturitas. 2023;179:107869.
7. Bliss ES, Wong RHX, Howe PRC, Mills DE. The Effects of Aerobic Exercise Training on Cerebrovascular and Cognitive Function in Sedentary, Obese, Older Adults. Frontiers in Aging Neuroscience. 2022;14.
8. Çetin E, Top EC, Şahin G, Özkaya YG, Aydin H, Toraman F. Effect of vitamin e supplementation with exercise on cognitive functions and total antioxidant capacity in older people. Journal of Nutrition, Health and Aging. 2010;14(9):763-9.
9. Chapman SB, Aslan S, Spence JS, DeFina LF, Keebler MW, Didehbani N, et al. Shorter term aerobic exercise improves brain, cognition, and cardiovascular fitness in aging. Frontiers in Aging Neuroscience. 2013;5(NOV).
10. Gaitán JM, Boots EA, Dougherty RJ, Oh JM, Ma Y, Edwards DF, et al. Brain Glucose Metabolism, Cognition, and Cardiorespiratory Fitness following Exercise Training in Adults at Risk for Alzheimer's Disease. Brain Plasticity. 2019;5(1):83-95.
11. Hötting K, Reich B, Holzschneider K, Kauschke K, Schmidt T, Reer R, et al. Differential cognitive effects of cycling versus stretching/coordination training in middle-aged adults. Health Psychology. 2012;31(2):145-55.
12. Huang J, Leng L, Hu M, Cui X, Yan X, Liu Z, et al. Comparative Effects of Different Exercise Types on Cardiovascular Health and Executive Function in Sedentary Young Individuals. Medicine and science in sports and exercise. 2025;57(6):1110-22.
13. Jonasson LS, Nyberg L, Kramer AF, Lundquist A, Riklund K, Boraxbekk CJ. Aerobic exercise intervention, cognitive performance, and brain structure: Results from the Physical Influences on Brain in Aging (PHIBRA) Study. Frontiers in Aging Neuroscience. 2017;8(JAN).
14. Langoni CDS, Resende TL, Barcellos AB, Cecchele B, Knob MS, Silva TDN, et al. Effect of Exercise on Cognition, Conditioning, Muscle Endurance, and Balance in Older Adults With Mild Cognitive Impairment: A Randomized Controlled Trial. Journal of geriatric physical therapy (2001). 2019;42(2):E15-E22.
15. Salmoirago-Blotcher E, Trivedi D, Dunsiger S, Harris K, Breault C, Yang Santos C, et al. Exploring Effects of Aerobic Exercise and Mindfulness Training on Cognitive Function in Older Adults at Risk of Dementia: A Feasibility, Proof-of-Concept Study. American Journal of Alzheimer's Disease and other Dementias. 2021;36.
16. Sink KM, Espeland MA, Castro Sweet CM, Church T, Cohen R, Dodson JA, et al. Effect of a 24-month physical activity intervention vs health education on cognitive outcomes in sedentary older adults: The LIFE randomized trial. JAMA. 2015;314(8):781-90.
17. Tarazona Santabalbina FJ, Gómez-Cabrera MC, Pérez-Ros P, Martínez-Arnau FM, Cabo H, Tsaparas K, et al. A Multicomponent Exercise Intervention that Reverses Frailty and Improves Cognition, Emotion, and Social Networking in the Community-Dwelling Frail Elderly: A Randomized Clinical Trial. Journal of the American Medical Directors Association. 2016;17(5):426-33.
18. Wilbur J, Miller AM, McDevitt J, Wang E, Miller J. Menopausal status, moderate-intensity walking, and symptoms in midlife women. Research and theory for nursing practice. 2005;19(2):163-80.
